# Evaluation of immunologic parameters in canine glioma patients treated with an oncolytic herpes virus

**DOI:** 10.20517/jtgg.2021.31

**Published:** 2021-12-05

**Authors:** M. R. Chambers, J. B. Foote, R. T. Bentley, D. Botta, D. K. Crossman, D. L. Della Manna, D. Estevez-Ordonez, J. W. Koehler, C. P. Langford, M. A. Miller, J. M. Markert, A. K. Olivier, N. B. Omar, S. R. Platt, D. R. Rissi, A. Shores, D. C. Sorjonen, E. S. Yang, A. B. Yanke, G. Y. Gillespie

**Affiliations:** 1 Department of Neurosurgery, University of Alabama at Birmingham (UAB), Birmingham, AL 35294, USA.; 2 Department of Microbiology, University of Alabama at Birmingham (UAB), Birmingham, AL 35294, USA.; 3 Department of Neurosurgery, College of Veterinary Medicine, Purdue University, West Lafayette, IN 47907, USA.; 4 Department of Genetics, University of Alabama at Birmingham (UAB), Birmingham, AL 35294, USA.; 5 Department of Radiation Oncology, University of Alabama at Birmingham (UAB), Birmingham, AL 35294, USA.; 6 Department of Pathobiology, College of Veterinary Medicine, Auburn University, Auburn, AL 36849, USA.; 7 Department of Comparative Pathobiology, College of Veterinary Medicine, Purdue University, West Lafayette, IN 47907, USA.; 8 Department of Pathology, College of Veterinary Medicine, Mississippi State University, Starkville, MS 39762, USA.; 9 Department of Neurosurgery, College of Veterinary Medicine, University of Georgia, Athens, GA 30602, USA.; 10 Athens Veterinary Diagnostic Laboratory, Department of Pathology, College of Veterinary Medicine, University of Georgia, Athens, GA 30602, USA.; 11 Department of Neurology & Neurosurgery, College of Veterinary Medicine, Mississippi State University, Starkville, MS 39762, USA.; 12 Department of Clinical Sciences, College of Veterinary Medicine, Auburn University, Auburn, AL 36849, USA.

**Keywords:** Oncolytic herpes virus, M032, canine glioma, large animal model, tumor microenvironment, NanoString

## Abstract

**Aim:**

To molecularly characterize the tumor microenvironment and evaluate immunologic parameters in canine glioma patients before and after treatment with oncolytic human IL-12-expressing herpes simplex virus (M032) and in treatment naïve canine gliomas.

**Methods:**

We assessed pet dogs with sporadically occurring gliomas enrolled in Stage 1 of a veterinary clinical trial that was designed to establish the safety of intratumoral oncoviral therapy with M032, a genetically modified oncolytic herpes simplex virus. Specimens from dogs in the trial and dogs not enrolled in the trial were evaluated with immunohistochemistry, NanoString, Luminex cytokine profiling, and multi-parameter flow cytometry.

**Results:**

Treatment-naive canine glioma microenvironment had enrichment of Iba1 positive macrophages and minimal numbers of T and B cells, consistent with previous studies identifying these tumors as immunologically “cold”. NanoString mRNA profiling revealed enrichment for tumor intrinsic pathways consistent with suppression of tumor-specific immunity and support of tumor progression. Oncolytic viral treatment induced an intratumoral mRNA transcription signature of tumor-specific immune responses in 83% (5/6) of canine glioma patients. Changes included mRNA signatures corresponding with interferon signaling, lymphoid and myeloid cell activation, recruitment, and T and B cell immunity. Multiplexed protein analysis identified a subset of oligodendroglioma subjects with increased concentrations of IL-2, IL-7, IL-6, IL-10, IL-15, TNFα, GM-CSF between 14 and 28 days after treatment, with evidence of CD4^+^ T cell activation and modulation of IL-4 and IFNγ production in CD4^+^ and CD8^+^ T cells isolated from peripheral blood.

**Conclusion:**

These findings indicate that M032 modulates the tumor-immune microenvironment in the canine glioma model.

## INTRODUCTION

### Glioma

Glioma is the most prevalent primary brain tumor in humans, accounting for approximately 30% of adults’ primary central nervous system tumors^[1,2]^. The disease poses a therapeutic challenge, with almost 25,000 gliomas diagnosed annually in the United States, resulting in disproportionate morbidity and mortality^[3]^. Despite decades of research and innumerable preclinical and clinical trials, progress has been extremely disappointing.

The molecular signatures of human glioma have been characterized by sequencing techniques. They are now being used to individualize treatments, and some biomarkers, such as IDH mutation, 1p19q co-deletion, MGMT promoter methylation, and EGFR vIII amplification, may be of prognostic value^[1,2]^. While the expanded use of whole-genome sequencing and targeted therapies have offered improved outcomes to some, the prognosis for the most malignant of these tumors remains dismal, with a median survival of approximately 17 months from diagnosis^[4]^. Indeed, advancements in brain tumor therapies have come slowly, and a comparative oncologic approach and modeling of immunotherapies offer great promise, with noted advantages and limitations^[5]^.

High-grade glioma resistance to treatment is attributed not only to tumor heterogeneity and invasiveness but also, perhaps most importantly, to active induction of immunosuppression by the tumor. Immunosuppressive cell populations have been identified within gliomas and in the TME^[6]^. The glioma TME is enriched primarily with tissue-resident, tumor-associated macrophages, which are the predominant immune cell population, myeloid-derived suppressor cells, and regulatory T cells^[7]^. Understanding the molecular basis for this immune-refractory state is key to identifying targets for therapy.

### Translational model

It is well established that the rodent glioma model differs in many ways from the spontaneously occurring human glioma^[8,9]^. In addition to sporadic development, the tumors in humans often have periods of latency, intra-tumoral heterogeneity, and more complex biology, with varied responses to therapy. Furthermore, most murine models lack an intact immune system^[10]^. Therapies validated in mouse models have failed to translate to successful outcomes in humans reliably^[11,12]^. Rodents have been and remain important models of human disease, but dogs represent an outbred natural model for the study of somatic mutations.

“*If one is developing translational models, the genetic and physiological similarities to humans are paramount*”^[13]^. The sporadic canine tumor model overcomes many of the rodent model deficiencies described here, and the pet dog with spontaneously occurring glioma may offer an ideal immune-competent model for cancer in humans. The clinical, anatomic, and histological similarities between canine and human cancer patients have been previously described^[14–16]^. Dogs respond to and similarly metabolize drugs to humans^[17,18]^. The incidence, pathogenesis, and factors affecting the progression of sporadically occurring gliomas have all been shown to be similar^[4,17–19]^. The natural lifespan of a dog is typically 5- to 8-fold shorter than that of humans, accelerating time to study results.

Pet dogs have incidences of spontaneous central nervous system tumors estimated at 14.5 per 100,000 animals, and up to 35% of spontaneously occurring CNS tumors in brachycephalic breeds are gliomas^[18]^. These present and behave much like gliomas in humans with similar radiographic, histopathologic, and genetic features. In the dog, high-grade oligodendrogliomas have been shown to be more similar to human glioblastoma multiforme than to human high-grade oligodendroglioma, typically lacking the favorable IDH mutation and 1p19q co-deletion^[20]^. They also respond to therapies much like their human counterparts^[20]^. Current standard of care for gliomas in both pet dogs and humans is a combination of maximum safe resection, chemotherapy, and radiation. Tumors typically recur, result in disability, and ultimately lead to premature death.

### The CANINE trial

Translational modeling and pre-clinical testing are essential to improve our understanding and guide the development of effective therapies. Pre-clinical testing of immune therapeutic approaches in large animal models may accurately inform human clinical trial design. To further examine the value of this approach, the CANine ImmunoNEurotherapeutics (“CANINE”) clinical trial funded through the Cancer Moonshot Program (NIH NCI/USPHS U01 CA224151–01) was designed to evaluate the safety and tolerability of a genetically engineered oncolytic herpes virus (oHSV M032) in pet dogs with sporadic gliomas, and to assess the immune response in pet dogs treated with intratumoral oncoviral therapy alone and in combination with oral checkpoint inhibition following maximum safe tumor resection^[6]^.

M032 is an HSV-1 virus genetically engineered to infect glial tumors, largely through binding oHSV to CD111 (nectin-1) expressed on the tumor cell’s surface. Deletion of both copies of the *γ*_*1*_*134.5* gene eliminates neurovirulence^[21–23]^. The virus is also armed with IL-12, a potent cytokine to elicit a robust immune response. M032 is highly antigenic; expresses IL-12; and the very high proportion of unmethylated CpG sequences in its DNA are readily recognized by toll-like receptors (TLR9) on immune-related cells, providing an adjuvant effect. As the virus infects and lyses tumor cells, the virus is released, and an immune response is stimulated. Longer-term results include innate immune cell activation, cytokine release, and cross-epitope spread, whereby T cells that initially recognized viral antigens begin to recognize tumor cell antigens. The M032-mediated expression of IL-12 completes the potent immune stimulation by providing local and regional activation of T cells. Targeted delivery of an oncolytic herpes virus expressing human IL-12 has been shown to modulate the human immune system, and human IL-12 has been shown in veterinary studies to induce a systemic T-cell response in dogs^[24,25]^.

As enrollment in the CANINE trial continues, we are examining the immune-modulatory effects of M032 on the tumor-immune microenvironment in canine glioma to identify biomarkers that might predict response to therapy.

## METHODS

### Trial design and oversight

“CANINE” is an ongoing phase 1 veterinary clinical trial described above. Between January 2018 and August 2020, 25 canine subjects with the presumptive diagnosis of glioma based on history, clinical examination, and MR imaging were screened and enrolled in the trial with informed consent from pet owners. Signalment and demographics were published with safety and interim survival data [Supplementary Table 1] and the 12-month schedule of inpatient and subsequent outpatient clinical, radiographic, and laboratory surveillance^[26]^. After treatment, one dog (007) left the study to undergo alternate therapies, and three dogs (015, 022, and 024) underwent tumor resection with catheter implantation but died of pneumonia prior to intracranial inoculation with the M032 virus. Based on data from 21 remaining dogs enrolled in Stage 1 of the trial, we demonstrated the safety and tolerability of the genetically modified herpes simplex virus. There were no adverse effects attributable to the virus and no dose-limiting toxicities. Stage 2 of the trial is enrolling now (*n* = 8) to evaluate response to combination treatment with surgery, oncolytic virus, and checkpoint inhibition.

This phase 1 canine clinical trial was approved by the UAB Institutional Animal Care and Use Committee (IACUC-21115) and by the respective IACUCs at participating regional veterinary study sites (Auburn University College of Veterinary Medicine IACUC 2018–3284, Mississippi State College of Veterinary Medicine IACUC-20–396, Purdue College of Veterinary Medicine PACUC 1812001831, and the University of Georgia College of Veterinary Medicine IACUC A2018 01–008-Y1-A0) to establish safety and tolerability. Dogs less than 6 months of age and those with a life expectancy of less than 6 months, as estimated by their respective primary care veterinarians and veterinary oncologists based on age at diagnosis, functional status at the time of presentation and comorbidities, were excluded. Those with tumors involving ventricles, basal nuclei, brainstem, or posterior fossa were also excluded.

According to the clinical trial protocol, pre-operative oral glucocorticoid administration was allowable if the dose did not increase within 2 weeks of surgery (indicating a clinically stable patient) and the total dose did not exceed 2 mg/kg per day of dexamethasone (or an equivalent dose of other glucocorticoids) at the time of enrollment. Importantly, most dogs referred to a tertiary care center for signs and symptoms of CNS disease, specifically brain tumors, receive empiric anti-inflammatory therapy with glucocorticoids prior to referral. In all cases, dogs that were receiving glucocorticoids at the time of referral were prescribed a weaning taper prior to surgery and with two exceptions, no dog was ever treated with dexamethasone (or dexamethasone equivalent) dose greater than 0.3 mg/kg po bid before enrollment and throughout the trial (006: 0.74 mg/kg po q 48 h preoperatively and 0.35 mg/kg po q 48 h postoperatively; 013: 0.84 mg/kg po bid preoperatively). All subjects that were not already therapeutically treated with an antiepileptic drug were treated with oral levetiracetam at a daily dose (30 mg/kg tid with Keppra or 30 mg/kg po bid with Keppra XR) for at least 6 weeks if there were no contraindications to its use. Treatment in all cases was scheduled within 2 weeks of enrollment.

Each dog underwent maximal safe resection of the tumor, as determined by the operating veterinary neurosurgeon. Twenty-two dogs had evaluable tumor tissue based on intraoperative biopsies [Table 1]. A Medtronic standard 23 mm translucent ventricular catheter (product #41115) was placed into the residual tumor following tumor resection. As oncotic pressure may push administration of a small volume of oHSV suspension into the peri-tumoral space away from the tumor in the interstitial fluid flow, accurate catheter placement was assured with direct visualization in each case for optimal virus delivery to tumor cells. The proximal end of the catheter was fixed to a percutaneously accessible reservoir (0.5 mL capacity) in the subcutaneous space beneath the scalp. Following closure, the fixed-length catheter was confirmed to be in an appropriate position via postoperative CT and/or MR imaging. As with intracranial catheters and subcutaneous reservoirs surgically placed in humans, once secured with the reservoir resting on the skull and following surgical closure, the implant has very little risk of displacement.

Following preliminary histological confirmation of diagnosis, intracranial inoculation with the M032 virus was performed via a single 1 mL infusion (0.5 mL of viral solution and 0.5 mL of sterile vehicle) through the subcutaneous reservoir. The viral dose delivered via catheter was determined via the previously published dose escalation scheme in subsequent cohorts of dogs. Based on the trial design, the virus dose was increased from 10^6^ to 10^9^ PFU in cohorts of three dogs each in the absence of any serious toxicity; once the highest safe dose was defined, ten additional dogs received the 10^9^ PFU dose to confirm the highest safe dose and complete Stage 1. Stage 2 continues, using the 10^9^ PFU dose with post-M032 infusion 30-day daily oral dose of indoximod, an Indoleamine-2,3 Dioxygenase inhibitor.

A secondary review of histopathology using NCI Comparative Brain Tumor Consortium (CBTC) Pathology Board criteria was completed by a panel of three board-certified veterinary pathologists (VPP) for final diagnoses^[14]^. The initial protocol proposed viral injection within 48 h of surgery to be certain there was time for at least a preliminary histopathologic diagnosis; however, this was modified to allow up to 7 days for recovery from surgery and tapering of glucocorticoid dosages and for final histopathologic diagnosis. The VPP secondary review resulted in a change in the final diagnosis in four cases. The time between surgery and administration of the virus is noted in Table 1.

### Samples

Biopsy (pre-M032 treatment) and necropsy (post-M032 treatment) specimens from subjects enrolled in the “CANINE” clinical trial and necropsy specimens from dogs not enrolled in the trial (A1–6) were obtained from formalin-fixed paraffin-embedded (FFPE) tumor. FFPE tumor tissue was used for immunohistochemical and NanoString mRNA gene expression analyses, as shown in Table 1.

Peripheral blood was collected from study subjects prior to surgery and at 2, 3, 14, 28, 90, 180, 270, and 365 days after surgery. In addition, peripheral blood from healthy control animals was obtained from canine blood donors at Auburn University, Mississippi State University, and the University of Georgia Colleges of Veterinary Medicine. In this interim report, we describe findings regarding those samples obtained at day 0 (pre-M032 treatment) and day 14 (post-M032 treatment). The remaining analysis of different time points is ongoing and not presented in this manuscript. All fresh anticoagulated blood from canine glioma patients and controls samples were separated by a Ficoll 1077 Hypaque gradient centrifugation, and the plasma and buffy coat containing the peripheral blood mononuclear cell (PBMC) fraction were collected. PBMCs were counted and then cryopreserved in DMEM/F12 medium + FBS (20%) and DMSO (7%) pending analysis.

### Flow cytometry

Cryopreserved PBMCs were recovered for FACS analysis and were pretreated by blocking in blocking buffer (1% FBS/PBS) with Human TruStain FcX Fc receptor blocking solution (BioLegend) per 1,000,000 cells. Cells were stained with the following fluorochrome-tagged antibodies, which have been validated to be specific or cross-reactive to canine immune cell epitopes, using manufacturer’s recommendations: (1) T and B cell panel: CD44-FITC (BioRad, YKIX337.78), CD5-PE (BioRad, YKIX322.3), CD79α-PerCP Cy5.5 (Invitrogen, clone HM47), CD4-PE Cy7 (BioRad, YKIX302.9), CD11b-PE CF594 (BioLegend, clone M1/70)^[27,28]^, CD21-AF647 (BioRad, CA2.1D6), CD45RA (BioRad, clone CA4.1D3), goat anti-mouse IgG APC Cy7 (BioLegend), CD8α AF700 (BioRad, clone YCATE55.9), CCR7 (BD, clone 150503)^[29]^, C45RA (BioRad, clone CA4.1D3), and Live/Dead Aqua (Invitrogen); and (2) T cell function panel: CD107β-FITC (BioRad, clone AC17), IL-4 PE (BioRad, clone CC02), CD4-PE Cy7 (BioRad, clone YKIX302.9), TNFα-PE CF594 (BioLegend, clone MaB11)^[30]^, IFNγ-AF647 (BioRad, clone CC302), CD8α-AF700 (BioRad, clone YCATE55.9), Granzyme B-Pacific Blue (BioLegend, clone GB11)^[31]^, and Live/Dead Aqua (Invitrogen). Cells were acquired on an LSR II. Flow cytometry gating was determined using cells stained with secondary only & single-color controls, and data were analyzed using FlowJo 10.

### Luminex multiplexed protein analysis

Cytokine and chemokine levels were quantified in plasma from subject whole blood using the caninespecific Milliplex multi-analyte panel kit CCYTOMAG-90K (Millipore-Sigma) and the MagPix instrument platform with related xPONENT software (Luminex Corporation). In addition, the readouts were analyzed with the standard version of EMD Millipore’s Milliplex Analyst software (Millipore-Sigma and Vigene Tech).

### *In vitro* PBMC stimulation

Cryopreserved PBMCs from healthy controls and canine glioma subjects (naïve and 14 days post M032 treatment) were thawed, and 10^6^ cells were plated into 24 well plates in 1 mL of RPMI media containing 10% FCS (HyClone), 2 mM L-glutamine (Thermo Fisher Scientific), and 50 U/mL Penicillin-Streptomycin (Thermo Fisher Scientific). Cells were stimulated with a 1× preparation of the eBioscience Cell Stimulation Cocktail containing PMA, ionomycin, and monensin (Invitrogen) for 5 h at 37 °C according to manufacturer’s instructions. Cells were then washed, surface stained with CD107B, CD4, and CD8, permeabilized with Cytoperm/Cytofix solution (BD), and then stained (intracytoplasmic) with antibodies specific to Granzyme B, IL-4, IFNγ, and TNFα. Specific information for those antibodies is described above (Flow Cytometry section) in the T cell function panel.

### Histology and immunohistochemistry

Initial biopsy (pre-M032 treatment) specimens were collected at the time of surgery. Necropsy (post-M032 treatment) specimens were collected from canine patients euthanized at one of the referring veterinary centers. Necropsy specimens were not available for all patients. Histologic diagnoses of low- or high-grade oligodendroglioma, astrocytoma, or undefined glioma were made by the attending veterinary pathologists at the respective veterinary medical centers using the CBTC Pathology Board criteria^[14]^. Diagnoses were then reviewed and modified as indicated based on evaluations of hematoxylin-eosin (HE), Olig2, and GFAP stained specimens by consensus of the VPP (JWK, DRR, JBF) following examination of all available tumor specimens.

Immunohistochemical analyses were performed at the University of Georgia College of Veterinary Medicine Comparative Pathology Laboratory utilizing diagnostically validated immunostaining assays for CD3, CD20, Iba1, factor VIII (FVIII)-related antigen, and Ki67. Conditions for each assay are summarized in Table 2.

For the characterization of the immune cell population, tissue sections were stained with anti-CD3, anti-CD20, and anti-Iba1; cumulative values were generated after the numbers of stained inflammatory cells were randomly counted in 2.37 mm^2^ as previously outlined^[32,33]^. Additional control tissues for CD3, CD20, and Iba1 included normal canine lymph node. Anti-FVIII-related antigen was utilized to assess the microvascular density within tumors. A cumulative value was generated after the number of stained capillaries was randomly counted in 2.37 mm^2^. The percentage of Ki67-positive neoplastic cells was calculated in 2.37 mm^2^ (equivalent to 10 FN22/40X fields), and a mean value was generated for each neoplasm.

Three canine patients with negative biopsies for tumor were used as internal controls for immunohistochemistry (IHC) and NanoString analyses. In each of these three cases, formalin-fixed, paraffin-embedded normal brain tissue was confirmed on the histopathologic assessment by the VPP. In addition, two (023 and 026) had no diagnostic lesions associated with any disease process, and the third (022) was diagnosed with granulomatous meningoencephalitis without tumor. These are referred to as controls for IHC and in the NanoString data set.

An additional five treatment-naive astrocytomas (A1–5 in Table 1) from subjects not enrolled in the clinical trial (contributed by D. Rissi at UGA) are included in immunohistochemical analyses (for a total of nine samples) in order to determine statistically significant changes in the expression of CD3, CD20, Iba1, factor VIII-related antigen, and Ki67 in the tumor microenvironment (TME).

### NanoString analyses

#### RNA isolation from FFPE tissue

To examine the molecular landscape of the canine glioma tumor microenvironment and determine neuroinflammation and cancer pathway gene expression, a novel custom Canine IO panel was employed [Supplementary Table 2]. The panel was developed specifically for use in canine cancer patients through a collaboration among members of the Pre-medical Cancer Immunotherapy Network for Canine Trials and supported by a grant from the National Cancer Institute (U24-CA224122 SUPPLEMENT; PI: Nicola Mason)^[34]^. Expression profiling of mRNA was performed on samples of 100 ng of RNA isolated from FFPE specimens on the nCounter system (NanoString Technologies) according to manufacturer’s instructions and analyzed using the nSolver analysis software (NanoString Technologies) and its built-in statistical analyses.

We performed NanoString-based mRNA expression profiling on paraffin-embedded tumor tissues using the Canine IO panel. Specimens included pre-M032 treatment (*n* = 19), matched post-M032 treatment (*n* = 6), controls (*n* = 3) and treatment-naïve astrocytomas from six dogs not enrolled in the trial (A1–6) shown in Table 3. Prior to the preparation of RNA, representative HE stained tissue sections were assessed to confirm the presence of tumor cells in the resected blocks. Only blocks containing tumor tissue were used for downstream NanoString analyses. For RNA isolation from FFPE tissue, three 20 μm-thick sections per region were deparaffinized by incubation in Xylene (2 × 10 min), 100% EtOH (10 min), 95% EtOH (10 min), 80% EtOH (10 min), and 75% EtOH (10 min) followed by a rinse in demineralized water. RNA from deparaffinized FFPE-tissue sections was extracted using the High Pure FFPET RNA Isolation Kit (Roche) according to the manufacturer’s protocols. RNA concentration and quality were determined using the Nanodrop system. Normalization, differential expression (DE), and pathway analyses were performed with Nanostring nCounter nSolver™ 4.0 (Nanostring MAN-C0019–08), along with Nanostring Advanced Analysis Module 2.0 plugin (Nanostring MAN-10,030–03) following the Nanostring Gene Expression Data Analysis Guidelines (Nanostring MAN-C0011–04).

### Statistical analyses

GraphPad Prism 9 was used for graphing data and statistical analyses (outlined in greater detail in figure legends). Briefly, for comparison of multiple columns of parametric data arranged in columns, a one-way analysis of variance (ANOVA) was performed using Sidak’s multiple comparison test to correct for statistical hypothesis testing, and for grouped parametric data, a two-way ANOVA was performed using Dunnet’s multiple comparison test to correct for statistical hypothesis testing (two-tailed *P* values < 0.05 indicative of statistical significance). For evaluation of statistical significance between two columns, an unpaired students *t* test (Welch’s correction was applied for samples with unequal variance) was used for direct comparison between two parametric data sets (two-tailed *P* values < 0.05 indicative of statistical significance). Advanced Analysis Module 2.0 software uses open-source R programs for QC, normalization, DE analysis, pathway scoring, and gene-set enrichment analysis.

## RESULTS

### Characterization of treatment-naive canine gliomas

**Figure 1.**
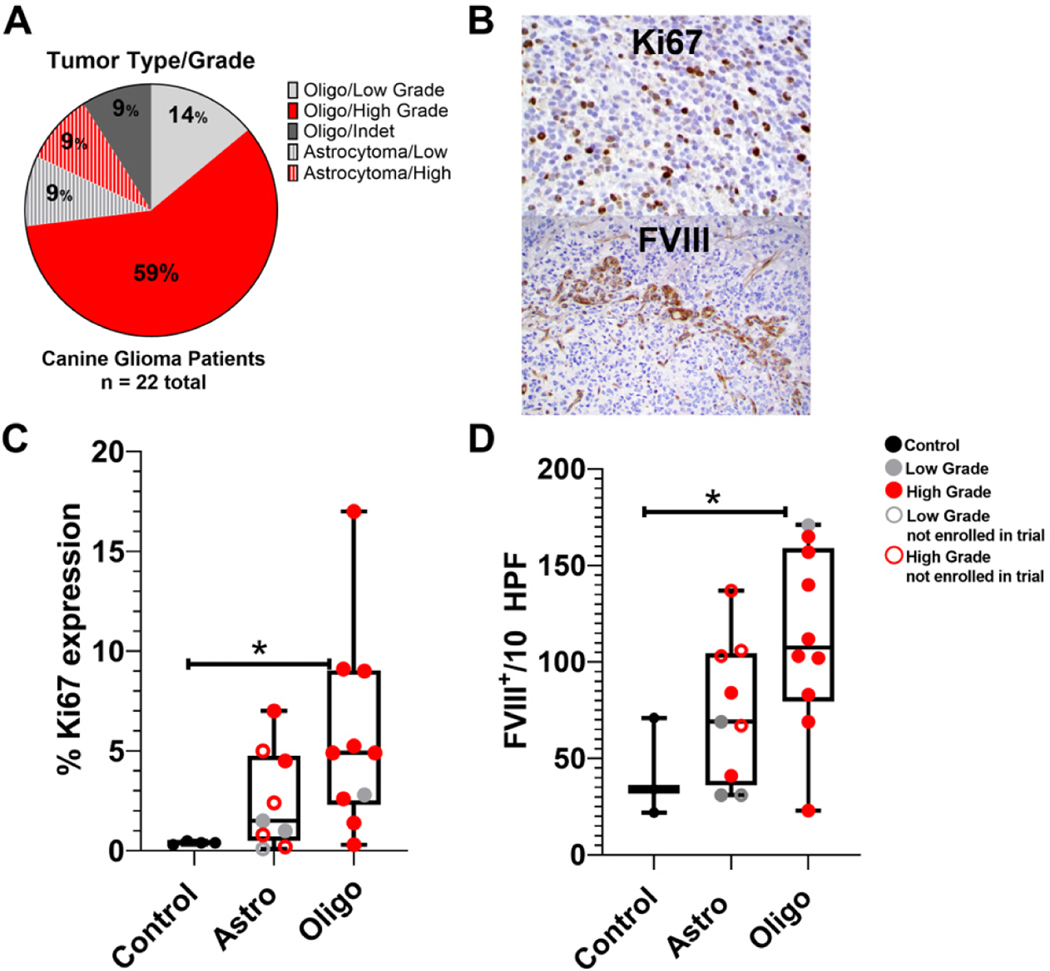
Tumor characteristics. (A) Clinical trial patient tumor types and grades. (B) Representative intra-nuclear Ki67 and FVIII-related antigen expression in canine glioma tumor microenvironment. (C) Percentages of Ki67 expression in controls (*n* = 3), astrocytomas {*n* = 9 [4 patient astrocytomas and 5 astrocytomas from dogs not in the clinical trial (A1–5)]}; and oligodendrogliomas (*n* = 10). (D) Numbers of FVIII-related antigen positive endothelial cells/10 cumulative high power fields in the same tumors. One-way analysis of variance (column analyses) with Sidak’s correction for multiple comparisons was applied with *P < 0.05 indicative of statistical significance for oligodendroglioma *vs.* control samples.

The majority of canine clinical trial patient gliomas included here exhibited morphologic and immunohistochemical features consistent with oligodendroglioma (13/18 high-grade, 3/18 low grade, and 2/18 undetermined grade after secondary review by the VPP). Conversely, astrocytomas comprised a smaller proportion (~21%) of consented subjects diagnosed with tumors (2/4 high-grade and 2/4 low grade) [Figure 1A].

Ki67 has been identified as a prognostic indicator in humans^[35]^. We performed characterization of Ki67 expression within canine tumor cells to evaluate tumor cell proliferation. Immunohistochemical analysis of the proportions of Ki67 expression in tumor cells in comparison to normal glial cells (normal control tissue from patients 022, 023, and 026) indicated increased Ki67 expression in tumor cells for both oligodendroglioma and astrocytoma; however, these trends were not statistically significant [Figure 1B and C].

Microvascular density was assessed in the canine glioma TME using immunohistochemistry for FVIII-related antigen. Canine oligodendrogliomas had significant increases in FVIII-related antigen expression in the TME, whereas increased expression in astrocytomas (*n* = 9) was not statistically significant [Figure 1B and D].

**Figure 2.**
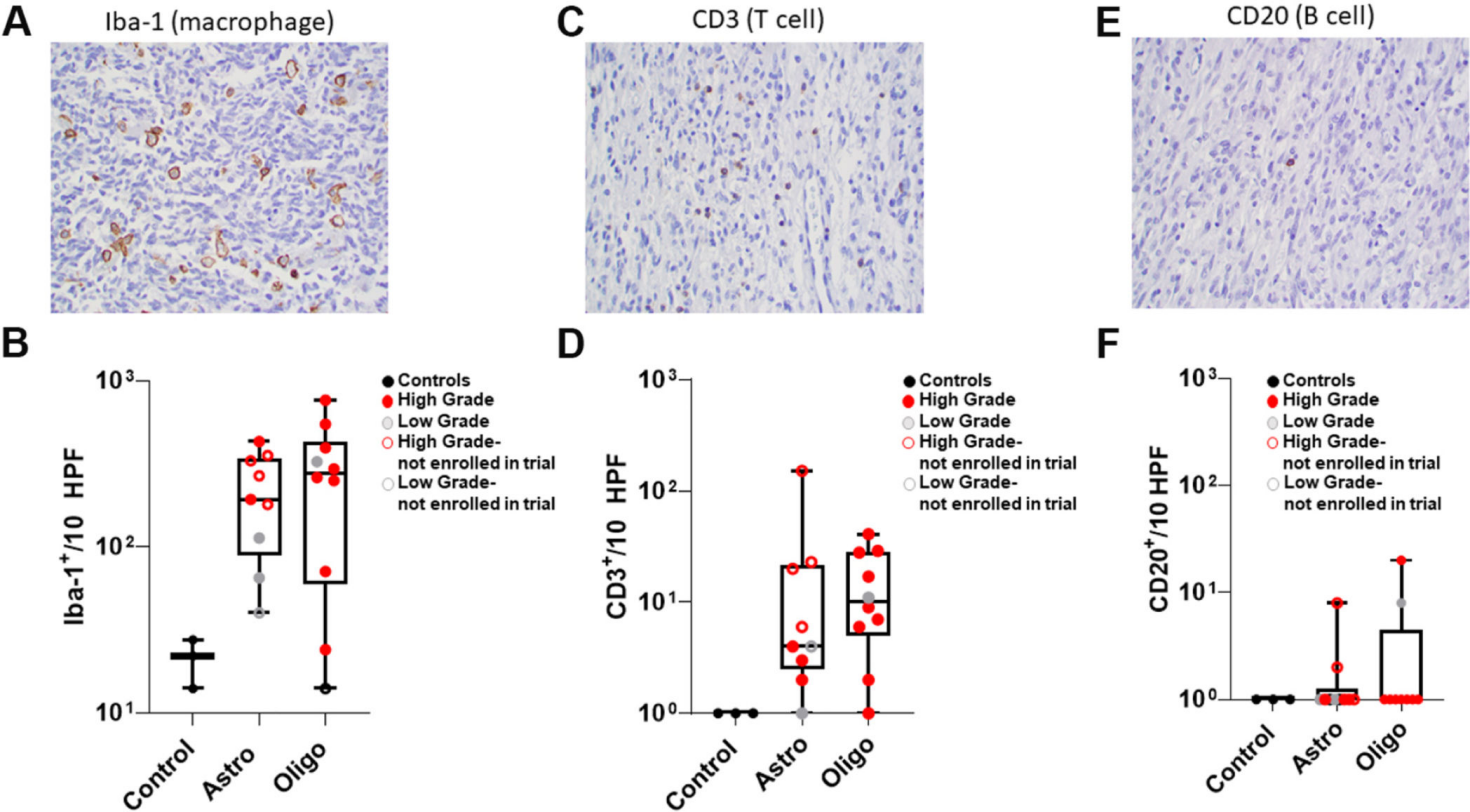
Immunostaining of treatment-naïve canine gliomas. (A, C, E) Representative immunostaining for (A) Iba1, (C) CD3, and (E) CD20 (40× magnification). (B, D, F) Numbers of (B) Iba1^+^ cells, (D) CD3^+^ lymphocytes, and (F) CD20^+^ lymphocytes within control (*n* = 3), astrocytoma (*n* = 9) and oligodendroglioma (*n* = 10) specimens. Using ordinary one-way ANOVA (column analyses) with Sidak’s multiple comparison test, statistical significance was calculated to correct the false discovery rate. Statistically significant differences were not noted despite obvious trends based on mean values for Astro and Oligo.

Immunohistochemistry for Iba1, CD3, and CD20 was performed on treatment-naïve canine astrocytomas (*n* = 9) and oligodendrogliomas (*n* = 10) to determine the density and distribution of macrophages (Iba1) and infiltrating T (CD3) and B lymphocytes (CD20) within the tumor microenvironment [Figure 2A, C and E]. Biopsies that did not have tumor present and were morphologically unremarkable on HE (022, 023, and 026) were used as controls. The tumor microenvironment for both astrocytomas and oligodendrogliomas were similarly highly enriched for Iba1^+^ macrophage (astrocytoma: 198 Iba-1+ cells/10 HPF; oligodendroglioma: 274 Iba-1+ cells/10 HPF), but not CD3^+^ T or CD20^+^ B cells, both of which were found in smaller numbers when present within the TME [Figure 2B, D and F].

### NanoString-based molecular characterization of the canine glioma TME before and after M032 treatment

Evaluation of differential gene expression between treatment-naïve canine gliomas and controls revealed a loose clustering of genetic signatures into four discernable groups, including those enriched for autophagy-related genes (Group 1), intrinsic signaling pathways potentially associated with tumorigenesis (Group 2), those bearing a mixture of tumor-related and immune gene signatures (Group 3), and those exhibiting an upregulation of immune mRNA signatures (Group 4) [Supplementary Figure 1].

**Figure 3.**
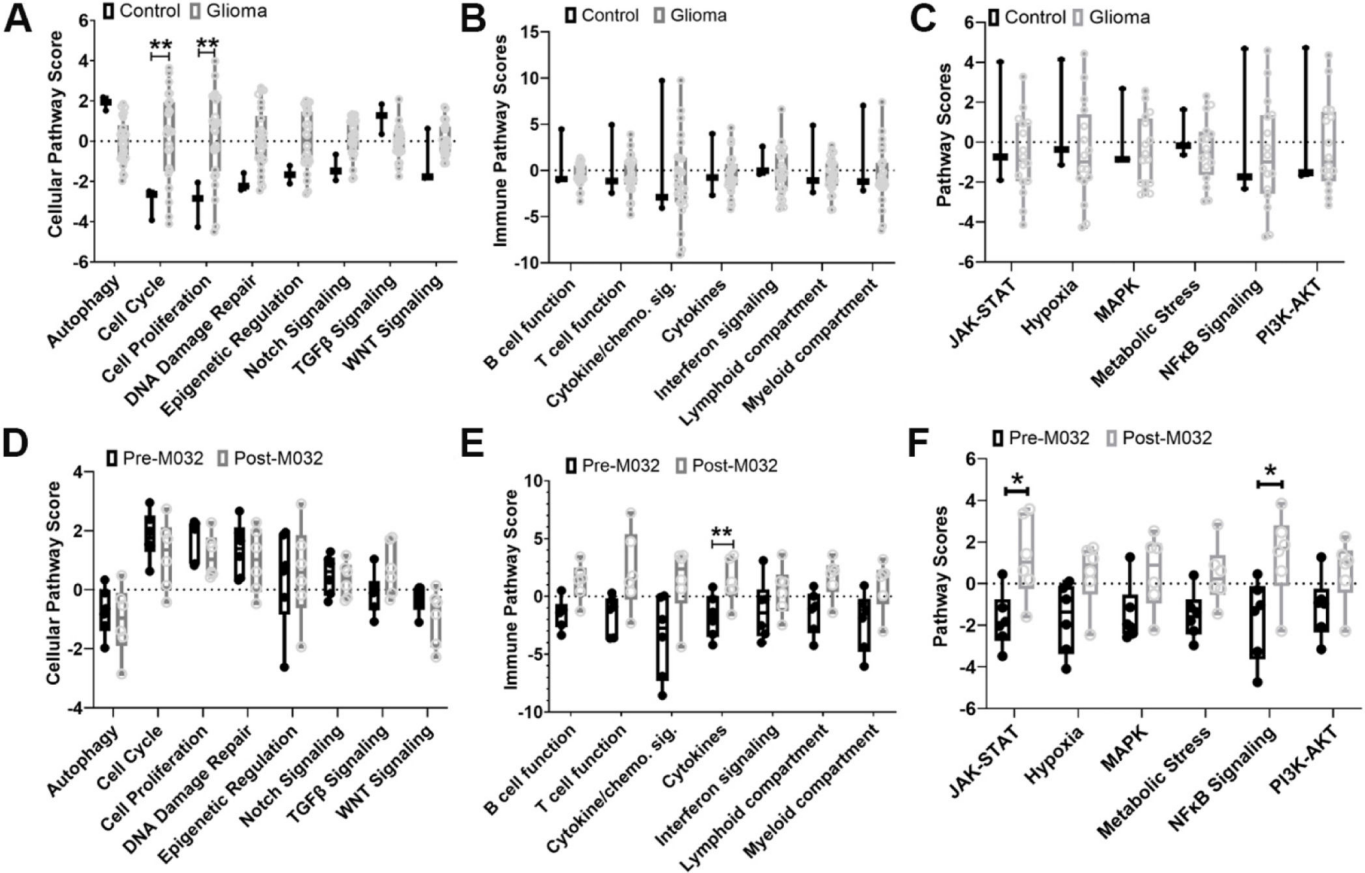
mRNA expression. Identification of differential expression of mRNA gene signatures and associated (A) cellular, (B) cell signaling, and (C) immune pathways, comparing initial biopsy specimens (*n* = 19) to brain tissue controls (*n* = 3). Identification of differential expression of mRNA gene signatures and associated with (D) cellular, (E) cell signaling, and (F) immune pathways, comparing the six matched patient samples before and after M032 treatment [Table 3]. Gene expression is grouped according to mRNA function and represented as pathway scores where > 0 indicates upregulation in tumor tissue and < 0 indicates downregulation in tumor tissue compared to normal. Statistical significance between groups was evaluated by two-way ANOVA with correction for multiple comparisons using Holm-Sidak *P < 0.05 and **P < 0.01.

Advanced analysis of the treatment-naïve patient glioma cohort revealed increased expression of mRNA associated with pathways potentially involved in cancer progression, including cell cycle, cellular proliferation, epigenetic regulation, Notch signaling, and DNA damage repair [Figure 3A, Supplementary Figure 1]. Analysis in this cohort revealed no significant differences in intracellular signaling or immune pathway scores compared to controls [Figure 3B and C]. However, in a small subset of treatment-naïve patient tumors, there was evidence for enrichment of immune pathways, likely indicating a variable degree of immune infiltration within the TME [Supplementary Figure 2].

Next, we analyzed differences in mRNA between treatment-naïve astrocytomas (008 and 009), oligodendrogliomas (005, 006, 010, 018), and matched specimens obtained at necropsy [Table 3]. For tumor signaling pathways, no significant differences between pre- and post-M032 treatment were noted [Figure 3D, Supplementary Figure 3]; however, analysis of these samples revealed differences in immune-related gene pathway scores [Figure 3E, Supplementary Figure 3] - specifically, scores for lymphoid and myeloid compartments, B cell and T cell function, cytokine and chemokine signaling, and cytokine expression. Analysis of mRNA associated with intracellular signaling pathways revealed significant differences in JAK-STAT and NFκB signaling pathways in post-M032 treated samples [Figure 3F]. Further analysis of mRNA expression associated with specific myeloid and lymphoid subsets revealed variable clustering with a small subset of patients (005 and 010) demonstrating an increase in NK and CD8^+^ T cells, and the remaining patients demonstrating variable degrees of enrichment for mRNA gene signatures associated with cytotoxic T and NK cells and other patients demonstrating enrichment of gene signatures specific for macrophages [Supplementary Figure 4]. After treatment with M032, a small increase in CD3^+^ T and CD20^+^ B cell infiltrate and decrease in % of tumor cells expressing Ki67 was noted [Supplementary Figure 5]; however, changes were not statistically significant. Additional IHC characterization of patient(s) TME post-M032 is pending.

These findings indicate an association between intratumoral injection of M032 and an mRNA transcriptomic pattern corresponding to the modulation of immune cells within the tumor microenvironment.

### Analysis of peripheral blood for correlates of tumor immune responses

**Figure 4.**
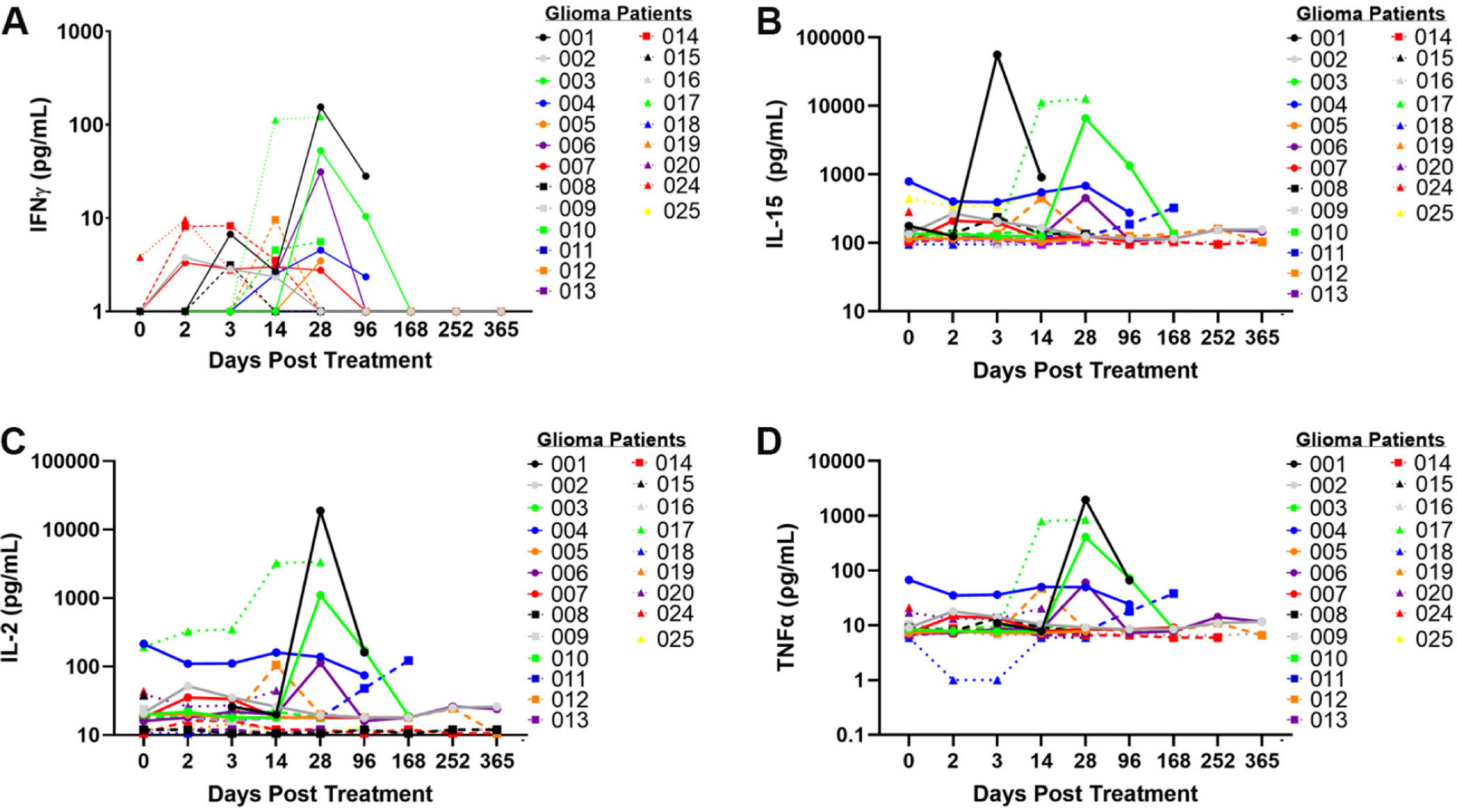
Expression of T_H_1 associated cytokines in canine glioma patients treated with M032. Expression of (A) IFNγ, (B) IL-15, (C) IL-2, and (D) TNFα prior to surgery (day 0), after surgery (days 2 and 3), and at various time points after intracranial infusion with M032 (days 14 through 365).

Immune function was characterized using multianalyte ELISA to determine cytokine and chemokine expression in peripheral blood and plasma obtained at specific time points after M032 treatment. Evaluation of T_H_1 associated cytokines (IFNγ, IL-15, IL2, and TNFα) revealed a transient increase in these cytokines in some patients between 14 and 28 days after viral infusion [Figure 4]. Similar increases in IL-6, IL-7, IL-10, IL-18, MCP-1, and GM-CSF between 14 and 28 days after viral infusion were noted [Supplementary Figure 6]. These findings demonstrate a correlation with M032 infusion and transient increases in cytokine and chemokine production between 14 and 28 days after M032 treatment.

**Figure 5.**
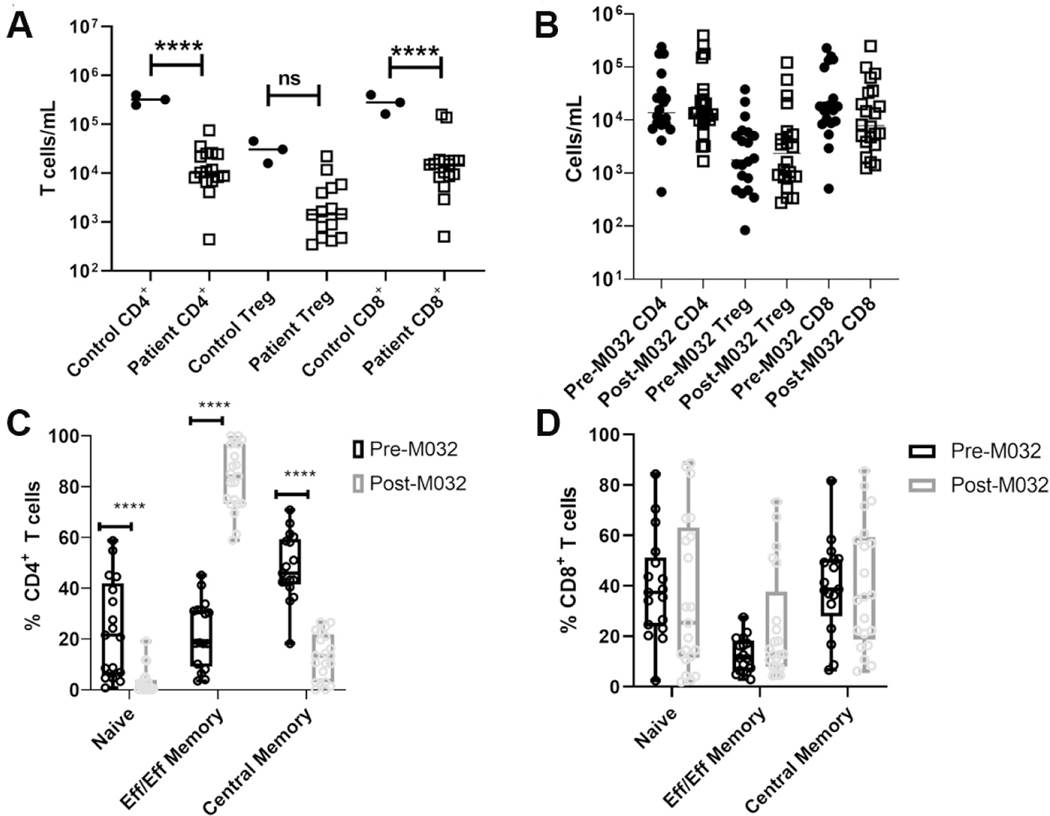
CD4^+^ T cells in canine glioma patients before and after treatment with M032. (A, B) Numbers of CD4^+^ Foxp3 ^−^, CD4^+^ Foxp3^+^ (Treg), and CD8^+^ T cells in the peripheral blood of (A) healthy controls *vs.* pre-M032 treatment canine glioma patients and (B) pre- *vs.* day 14 post-M032 treatment canine glioma patients. (C, D) The proportions of naïve (CD44^lo^ CD45RA^hi^), central memory (CD44^hi^ CD45RA^hi^), and effector/effector memory (CD44^hi^ CD45RA^lo^) in (C) CD4^+^ and (D) CD8^+^ T cells. Two-way ANOVA for grouped analyses and Dunnet’s correction for multiple comparisons was used to calculate statistical significance. ****P < 0.0001, ***P < 0.001, **P <0.01.

To determine if the increase is in T_H_1 cytokines within the plasma between 14 and 28 days after treatment with M032 correlated with activation of cytotoxic and T_H_1 effector responses in canine glioma patients, we evaluated the phenotypes and functions of recirculating T cells 14 days after M032 treatment using multiparameter flow cytometry [Supplementary Figure 7]. Analysis of PBMCs prior to M032 treatment revealed significantly diminished numbers of CD4^+^ and CD8^+^ T cells compared to normals [Figure 5A]. In addition, minimal changes were noted in T cell numbers at 14 days post-M032 [Figure 5B]; however, there was a significant increase in the proportion of CD44^hi^ CD45RA^lo^ CD4^+^ T cells and a similar trend in CD44^hi^ CD45RA^lo^ CD8^+^ T cells [Figure 5C and D] 14 days after M032 treatment.

**Figure 6.**
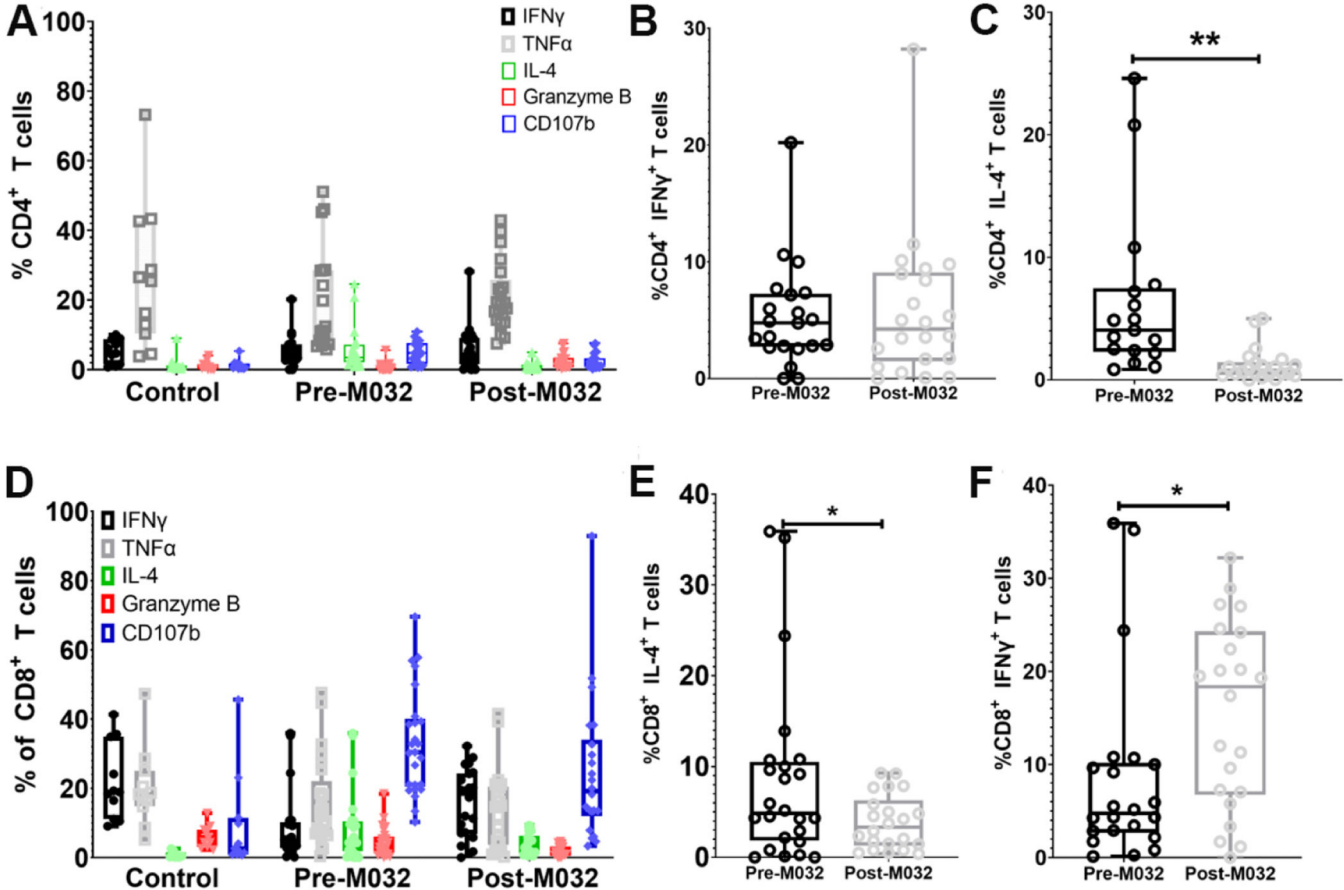
IL-4 and IFNγ production in recirculating T cell subsets in canine glioma patients before and after treatment with M032. (A) Percentages of IFNγ, TNFα, IL-4, Granzyme B, and CD107 expression in CD4^+^ T cells after PMA ionomycin stimulation in healthy controls, canine glioma patients pre-M032 treatment, and at day 14 post-M032 treatment. Comparison between pre-M032 (black) and day 14 post-M032 (gray) revealed (B) no significant change in IFNγ and (C) a significant decrease in IL-4 post-M032 treatment. (D) Percentages of IFNγ, TNFα, IL-4, Granzyme B, and CD107B expression in CD8^+^ T lymphocytes after PMA ionomycin stimulation in healthy controls, pre-M032 treatment canine glioma patients and day 14 post-M032 treatment patients. Comparison between pre-M032 (black) and day 14-post M032 (gray) revealed a significant (E) decrease in IL-4 and (F) increase in IFNγ production at 14 days after treatment. Statistical significance (B-E) was evaluated using an unpaired student’s *t* test (Welch’s correction for unequal variance). *P < 0.05, **P < 0.01, ***P < 0.001, ****P < 0.

Next, we evaluated the function of CD4^+^ and CD8^+^ T cells 5 h after *in vitro* stimulation with PMA and ionomycin in the presence of the intracellular transport inhibitor, monensin [Supplementary Figure 8]. Minimal differences were noted in IFNγ, TNFα, Granzyme B, and CD107B expression when comparing CD4^+^ T cells from glioma patients before and after M032 treatment [Figures 6A and B]; however, a significant reduction in IL-4 production between pre- and post-M032 treatment in peripheral blood CD4^+^ T cells was noted [Figure 6C]. Evaluation of CD8^+^ T cells revealed minimal differences in TNFα, CD107, and Granzyme B before and after M032 treatment [Figure 6D]; There was a significant decrease in IL-4 and an increase in IFNγ within the CD8^+^ T cell population after M032 stimulation [Figure 6E and F]. These findings indicate immune modulation in peripheral T cell subsets, specifically a shift from T_H_2 (IL-4) to T_H_1 (IFNγ) cytokine production within recirculating T cells after M032 treatment. These findings suggest a correlation between M032 treatment and peripheral T cell activation and modulation of IL-4 and IFNγ production.

## DISCUSSION

Here, we present interim data from “CANINE” (“CANine ImmunoNEurotherapeutics”), an ongoing phase 1 clinical trial. Findings suggest that intratumoral treatment of canine gliomas with the oncolytic herpes simplex virus M032 modulates immune responses systemically and within the tumor microenvironment. The focus of this interim examination of the immune-modulatory effects of oHSV M032 is viral-infusion-induced changes in the TME and periphery consistent with priming innate and adaptive immune responses.

The majority of canine gliomas enrolled in this trial were high-grade oligodendrogliomas, consistent with our current understanding of the glioma landscape in pet dogs^[18]^. Similar to human high-grade gliomas, increased microvascular proliferation in canine gliomas correlated with higher-grade^[13]^. The untreated canine glioma microenvironment for oligodendroglioma and astrocytoma was highly enriched for Iba1+ macrophages, and microglial cells but not CD3^+^ T or CD20^+^ B cells, both of which were found in very small numbers within the TME consistent with previous findings in canine glioma patients analyzed at necropsy^[36–39]^. Our findings are consistent with these studies and with what is seen in human glioblastoma multiforme, confirming the immunologically “cold” nature of these tumors^[7,40]^. Ideally, immunotherapies transform these tumors from immunologically “cold” to “hot”. Immune adjuvants including CpG, STING agonists, oncolytic dsDNA, and RNA viruses induce production of type I interferons leading to innate immune activation, priming, and recruitment of cytotoxic T cells and a resurgence of immune surveillance in immunologically cold tumors^[41]^.

NanoString analyses of treatment-naïve tumors revealed enrichment for tumor intrinsic pathways consistent with suppression of tumor-specific immunity and support of tumor progression in both astrocytomas and oligodendrogliomas, with increased copy numbers of mRNA for cell cycle proteins (CCND1, CDKN2C, CDK1, CDK4, MK167, and RAD51; Supplementary Table 3), DNA damage repair (p53), epigenetic regulation (Olig2, SOX10, and ESR1), MAPK signaling (ERBB3, RAF1, NRAS, MAP3K1), angiogenesis (PDGFRA), Notch and WNT signaling.

M032 induced intratumoral mRNA transcription signatures indicative of immune modulation in 83% (5/6) of canine glioma patients, including increased copy number of mRNAs [based on the top 20 differentially expressed genes] corresponding with interferon signaling (IFNA7 and IL29L), lymphoid and myeloid cell activation, recruitment (L selectin, IL-16, TLR9), and T and B cell immunity (AICDA). In addition, an increase in mRNAs associated with PI3K-AKT, MAPK, NFκB, and specific mRNA for proteins involved in immunosuppression (PD-1, B7-H3, PVR, and NT5E) may imply adaptation (animals succumbed to advanced disease). These findings indicate that M032 provokes an inflammatory gene signature within these tumors [Supplementary Tables 3 and 4].

Immune responses in peripheral blood were examined to identify biologic correlates associated with M032-induced changes in the TME. We noted increased cytokine and chemokine production between 14 and 28 days after treatment with M032 in 20%−30% of the canine patients; that is, for each cytokine evaluated, 20%−30% of patients exhibited a peak in this cytokine between days 14 and 28. Increases in IFNγ, IL-15, and IL-2 are suggestive of T_H_1 and cytotoxic immune responses. We see significant increased CD4^+^ T cell activation, corresponding decreases in IL-4, and increases in IFNγ production in T cells. Interestingly, we did not see increases in numbers of CD4^+^ and CD8^+^ T cells in the peripheral blood prior to M032 treatment. We did see the temporal elevation of T_H_1 cytokines and other inflammatory cytokines in a subset of canine oligodendroglioma subjects following treatment, specifically between 14 and 28 days after viral therapy.

It is unknown how long M032 persists in the TME; however, adaptive immune responses continue to build over weeks after the challenge. With regularly scheduled assessments testing for the presence of viral dsDNA in peripheral tissues (saliva and blood) and no evidence of peripheral dissemination of the virus, we believe that these findings indicate an M032-driven response predominantly in the TME. Interim analysis of T cell activation and cytokine production provides evidence of immune modulation in peripheral T cell subsets following treatment with oHSV M032. Oncolytic viral therapies have demonstrated success in priming adaptive immune responses and synergy with PD-1 immune checkpoint blockade in the preclinical and clinical settings^[42–45]^. In these trials, activation of peripheral T cells was noted^[46]^.

Oncolytic viral therapy is an emerging, unique and effective immune-based weapon against cancer. The ability to engineer viruses to tame their neurovirulence while enhancing their ability to infect and kill tumor cells has proven a major asset to their study. These viruses have the ability to recruit and potentiate innate immune responses, which have often been suppressed by the tumors, by inducing type I interferon production^[47,48]^, priming of innate and adaptive immune responses^[49]^, and antigenic cross-epitope spread^[50]^.

Limitations in this study include the many potentially confounding variables associated with clinical trials, such as the timing of treatment, patient (breed) and surgical variables, differences in tumor volumes, and the clinical use of potentially immunosuppressive dosages of corticosteroids. Human studies have recently described the detrimental effects of dexamethasone on the number and functional capacities of effector immune cells^[51,52]^. Recent findings regarding the potential effects of intraperitoneal corticosteroids on immunotherapy in a mouse model of GBM have also been noted^[53]^. We agree with these authors as they suggest that much work remains to reliably correlate the effects of peri-operative steroids and response to immunotherapies. However, we cannot discount the role of low-dose corticosteroids in this process. In addition, it has been demonstrated that intracranial tumors (GBM) influence S1P1R-mediated T cell recirculation through an unknown mechanism independent of corticosteroid usage^[54]^. In these pet dogs, treatment is provided as indicated for best care by their primary care veterinarians prior to referral and enrollment. In every case treated for presumed symptomatic cerebral edema, steroids were given at the lowest dose and duration possible at the discretion of the treating veterinarians and in the best interest of the pet dogs. We will further examine the effects of corticosteroid use on immune modulation and survivals in this canine clinical trial. Conclusions are tempered as well by the small number of samples, the interim nature of the findings, and the inability to serially sample changes in the tumor microenvironment.

This work further validates the spontaneous canine glioma model as a useful model for translational studies of the disease in humans. We have demonstrated an intratumoral immunomodulatory effect in canine gliomas following treatment with surgery, systemic steroids, and M032, a genetically modified oncolytic herpes virus, with a subsequent increased interferon response, production of chemokines, and signs of increased tumor-infiltrating lymphocyte activity. Compensatory immune-suppressive pathways were also noted to be upregulated, suggesting adaptive mechanisms of immune suppression [Supplementary Figure 3]. For a large animal comparative oncology approach to provide maximum information to accelerate the clinical translation of next generation immunotherapies and identify correlative biomarkers of therapeutic response, it is necessary to develop and employ research tools for deep interrogation of the immune response. The location and size of many tumors from animal models make isolation of sufficient tissue for in-depth immunologic and molecular characterization a considerable challenge. Here, we include NanoString technology, including the first use of a novel Nanostring Canine IO panel to evaluate indicators of tumor progression and immune responses before and after treatment on formalin-fixed, paraffin-embedded tumor tissue. The Canine IO panel contains genes selected based on their relevance for the study of oncology and expression profiles (from both RNA-Seq and nCounter experiments) and known to be associated with intracranial gliomas in both pet dogs and humans^[34]^.

Given the immunosuppressive nature of these tumors, it is logical to expect combinations of therapies to improve outcomes. Targeted delivery of M032, an oncolytic herpes virus expressing human IL-12, may serve as one effective component in a combination immunotherapy approach to treating gliomas. The findings from this study support further clinical trials using combination therapies with M032. The CANINE clinical trial and a concurrent adult human clinical trial both using cGMP M032 are ongoing (NCT02062827). In addition, a Phase I trial in adults with a chimeric, unarmed oHSV, C134, is ongoing (NCT03657576). In children with brain tumors, a Phase 1 trial of an unarmed oHSV, G207, has been reported (NCT03911388), and a Phase 2 clinical trial in children with un-armed G207 oHSV will begin in the Fall (NCT04482933)^[55]^. Larger-scale randomized trials may be needed to examine the benefits of this comparative approach critically. We will continue to investigate these exciting findings and patterns in the larger cohort of post-M032 treated canine glioma patients in the CANINE trial.

## Supplementary Material

supplementary materials

## Figures and Tables

**Table 1.** Canine patient and tumor characteristics

Patient	Prelim (final) tumor diagnosis	Prelim (final) tumor grade	Time from surgery to treatment, days	Survival, following treatment, days	M032 dose	Ki67	FVIII-ra	Iba-1	CD3	CD20
001	Oligo	Low	1	108	10^6^	−	−	−	−	−
002	Oligo	High	1	372	10^6^	−	−	−	−	−
003	Oligo	High	1	232	10^6^	5.25	102	251	4	0
004	Astro	Low	1	169	10^7^	1	69	65	2	0
005^N^	Oligo	High	1	63	10^7^	2.6	140	549	9	0
006^N^	^+^Astro (Oligo)	Indet (High)	2	415	10^7^	4.9	103	262	7	0
007	Oligo	Indet	0	**	10^8^	−	−	−	−	−
008^N^	^+^Oligo (Astro)	High	1	41	10^8^	7	41	431	3	0
009^N^	Astro	High	7	11	10^8^	4.5	31	193	4	1
010^N^	Oligo	High	8	43	10^9^	4.9	69	765	41	6
011	Oligo	High	1	223	10^9^	−	−	−	−	−
012	Oligo	Indet	6	612	10^9^	−	−	−	−	−
013	Oligo	High	1	43	10^9^	9	112	24	17	0
014	Oligo	Low	7	629	10^9^	2.8	157	71	11	8
015	^+^Oligo	Low (High)	N/A	N/A	N/A	1.4	171	326	0	0
016	Astro	Low	6	425	10^9^	1.5	84	113	1	0
017	Oligo	Low	7	28	10^9^	−	−	−	−	−
018^N^	Oligo	High	3	70	10^9^	−	−	−	−	−
019	^+^GBM (Oligo)	High	3	62	10^9^	−	−	−	−	−
020	Oligo	High	6	22	10^9^	17	165	395	29	0
022	GME	N/A	N/A	N/A	N/A	0.4	25	72	0	0
023	Indet (Gliosis)	Indet (N/A)	N/A	N/A	10^9^	1.2	14	34	0	0
024	^+^GBM (Oligo)	High	2	2	N/A	0.3	23	14	0	0
025	^+^Astro (Oligo)	High	1	80	10^9^	9.1	83	295	4	2
026	Normal	N/A	N/A	*	N/A	5	N/A	22	0	0
A1	Astro	Low	N/A	N/A	N/A	5	67	355	20	0
A2	Astro	High	N/A	N/A	N/A	0.8	137	268	152	8
A3	Astro	High	N/A	N/A	N/A	2.4	106	180	23	0
A4	Astro	High	N/A	N/A	N/A	0.2	103	333	6	0
A5	Astro	Low	N/A	N/A	N/A	0.1	31	40	4	0
A6	Astro	Indet	N/A	N/A	N/A	−	−	−	−	−

Dog 021 was excluded after final diagnosis was modified to meningioma.

+ Indicates that the final diagnosis was later modified by the veterinary pathologists

N matching post-mortem tumor specimen collected and available for immunohistochemistry and Nanostring

* still living

** left study/lost to follow up

−: not available. Prelim: Preliminary; Oligo: oligodendroglioma; Astro: astrocytoma; GBM: glioblastoma multiforme; A1–6: necropsy specimens from dogs not enrolled in the trial; N/A: not applicable; Indet: indeterminate grade; FVIII-ra: factor VIII-related antigen.

**Table 2.** Primary antibodies used for immunohistochemistry

Antigen	Antibody name	Supplier/Cat#	Dilution	Pretreatment conditions	Target cell population
CD3	Rabbit polyclonal	Dako/A05452	1:1000	Citrate buffer-15min @110C	T cells
CD20	Rabbit polyclonal	Biocare/121R-18	1:2000	None	B cells
Iba-1	Rabbit polyclonal	Wako/019-19741	1:8000	Citrate buffer-15min @110C	Microglia macrophage
FVIII	Rabbit polyclonal	Cell Marque/250A-18	RTU	Citrate buffer-15min @110C	Endothelium
Ki67	Rabbit monoclonal antibody	Cell Marque/275R-18	RTU	Citrate buffer-15min @110C	Proliferating tumor cells

The antigen, antibody, supplier, dilution, pretreatment conditions, and cell target for labeling are indicated for this immunohistochemical study.

**Table 3.** Samples included in NanoString analyses

Patient	Intraoperative biopsy	Necropsy tissue	Final diagnosis	Grade	M032 dosage, PFU	Survival, days
001	+	−	Oligo	Low	10^6^	108
002	+	−	Oligo	High	10^6^	372
005	+	+	Oligo	High	10^7^	63
006	+	+	Oligo	High	10^7^	415
007	+	−	Oligo	Indet	10^8^	**
008	+	+	Astro	High	10^8^	41
009	+	+	Astro	High	10^8^	11
010	+	+	Oligo	High	10^9^	43
012	+	−	Oligo	Indet	10^9^	612
013	+	−	Oligo	High	10^9^	43
014	+	−	Oligo	Low	10^9^	629
015	+	−	Oligo	High	N/A	N/A
016	+	−	Astro	Low	10^9^	425
018	+	+	Oligo	Low	10^9^	70
019	+	−	Oligo	High	10^9^	62
020	+	−	Oligo	High	10^9^	22
022	+	−	GME	N/A	N/A	N/A
023	+	−	No tumor	N/A	N/A	N/A
024	+	−	Oligo	High	10^9^	2
025	+	−	Oligo	High	10^9^	80
026	+	−	No tumor	N/A	N/A	*
A1	−	+	Astro	Low	N/A	N/A
A2	−	+	Astro	High	N/A	N/A
A3	−	+	Astro	High	N/A	N/A
A4	−	+	Astro	High	N/A	N/A
A5	−	+	Astro	Low	N/A	N/A
A6	−	+	Astro	Indet	N/A	N/A

+: Available

−: not available

* still living

** left study/lost to follow up; N/A: not applicable; Indet: indeterminate.

## References

[1] WellerM, WickW, AldapeK, Glioma. Nat Rev Dis Primers 2015;1:15017. DOI PubMed2718879010.1038/nrdp.2015.17

[2] WenPY, KesariS. Malignant gliomas in adults. N Engl J Med 2008;359:492–507. DOI PubMed1866942810.1056/NEJMra0708126

[3] SiegelRL, MillerKD, FuchsHE, JemalA. Cancer statistics, 2021. CA Cancer J Clin 2021;71:7–33. DOI PubMed3343394610.3322/caac.21654

[4] OstromQT, PatilN, CioffiG, WaiteK, KruchkoC, Barnholtz-SloanJS. CBTRUS statistical report: primary brain and other central nervous system tumors diagnosed in the United States in 2013–2017. Neuro Oncol 2020;22:iv1-iv96. DOI PubMed PMC10.1093/neuonc/noaa200PMC759624733123732

[5] ParkJS, WithersSS, ModianoJF, Canine cancer immunotherapy studies: linking mouse and human. J Immunother Cancer 2016;4:97. DOI PubMed PMC2803182410.1186/s40425-016-0200-7PMC5171656

[6] ChambersMR, BentleyRT, CrossmanDK, The one health consortium: design of a phase I clinical trial to evaluate M032, a genetically engineered HSV-1 expressing IL-12, in combination with a checkpoint inhibitor in canine patients with sporadic high grade gliomas. Front Surg 2020;7:59. DOI PubMed PMC3300562310.3389/fsurg.2020.00059PMC7484881

[7] DeCordovaS, ShastriA, TsolakiAG, Molecular heterogeneity and immunosuppressive microenvironment in glioblastoma. Front Immunol 2020;11:1402. DOI PubMed PMC3276549810.3389/fimmu.2020.01402PMC7379131

[8] KijimaN, KanemuraY. Mouse models of glioblastoma. In: De VleeschouwerS, editor. Glioblastoma. Brisbane (AU): Codon Publications; 2017. p. 131–9. DOI29251866

[9] LentingK, VerhaakR, Ter LaanM, WesselingP, LeendersW. Glioma: experimental models and reality. Acta Neuropathol 2017;133:263–82. DOI PubMed PMC2807427410.1007/s00401-017-1671-4PMC5250671

[10] MiyaiM, TomitaH, SoedaA, YanoH, IwamaT, HaraA. Current trends in mouse models of glioblastoma. J Neurooncol 2017;135:423–32. DOI PubMed PMC2905280710.1007/s11060-017-2626-2PMC5700231

[11] PerelP, RobertsI, SenaE, Comparison of treatment effects between animal experiments and clinical trials: systematic review. BMJ 2007;334:197. DOI PubMed PMC1717556810.1136/bmj.39048.407928.BEPMC1781970

[12] U.S. Department of Health and Human Services, FDA. Challenge and opportunity on the critical path to new medical technologies. March, 2004. Available from: https://www.who.int/intellectualproperty/documents/en/FDAproposals.pdf [Last accessed on 21 Oct 2021].

[13] AlvarezCE. Naturally occurring cancers in dogs: insights for translational genetics and medicine. ILAR J 2014;55:16–45. DOI PubMed2493602810.1093/ilar/ilu010

[14] KoehlerJW, MillerAD, MillerCR, A revised diagnostic classification of canine glioma: towards validation of the canine glioma patient as a naturally occurring preclinical model for human glioma. J Neuropathol Exp Neurol 2018;77:1039–54. DOI PubMed PMC3023991810.1093/jnen/nly085PMC6181180

[15] RichardsKL, Motsinger-ReifAA, ChenHW, Gene profiling of canine B-cell lymphoma reveals germinal center and postgerminal center subtypes with different survival times, modeling human DLBCL. Cancer Res 2013;73:5029–39. DOI PubMed PMC2378357710.1158/0008-5472.CAN-12-3546PMC3755352

[16] FengerJM, LondonCA, KisseberthWC. Canine osteosarcoma: a naturally occurring disease to inform pediatric oncology. ILAR J 2014;55:69–85. DOI PubMed2493603110.1093/ilar/ilu009

[17] HicksJ, PlattS, KentM, HaleyA. Canine brain tumours: a model for the human disease? Vet Comp Oncol 2017;15:252–72. DOI PubMed2598867810.1111/vco.12152

[18] PriesterWA, McKayFW. The occurrence of tumors in domestic animals. Natl Cancer Inst Monogr 1980;(54):1–210. PubMed7254313

[19] SnyderJM, ShoferFS, WinkleTJ, MassicotteC. Canine intracranial primary neoplasia: 173 cases (1986–2003). J Vet Intern Med 2006;20:20–75. DOI PubMed1673410610.1892/0891-6640(2006)20[669:cipnc]2.0.co;2

[20] MillerAD, MillerCR, RossmeislJH. Canine primary intracranial cancer: a clinicopathologic and comparative review of glioma, meningioma, and choroid plexus tumors. Front Oncol 2019;9:1151. DOI PubMed PMC3178844410.3389/fonc.2019.01151PMC6856054

[21] Estevez-OrdonezD, ChagoyaG, SalehaniA, Immunovirotherapy for the treatment of glioblastoma and other malignant gliomas. Neurosurg Clin N Am 2021;32:265–81. DOI PubMed3378150710.1016/j.nec.2020.12.008PMC8519502

[22] ChouJ, KernER, WhitleyRJ, RoizmanB. Mapping of herpes simplex virus-1 neurovirulence to gamma 134.5, a gene nonessential for growth in culture. Science 1990;250:1262–6. DOI PubMed217386010.1126/science.2173860

[23] FriedmanGK, BernstockJD, ChenD, Enhanced sensitivity of patient-derived pediatric high-grade brain tumor xenografts to oncolytic HSV-1 virotherapy correlates with nectin-1 expression. Sci Rep 2018;8:13930. DOI PubMed PMC3022476910.1038/s41598-018-32353-xPMC6141470

[24] NguyenHM, Guz-MontgomeryK, SahaD. Oncolytic virus encoding a master pro-inflammatory cytokine interleukin 12 in cancer immunotherapy. Cells 2020;9:400. DOI PubMed PMC10.3390/cells9020400PMC707253932050597

[25] PavlinD, CemazarM, SersaG, TozonN. IL-12 based gene therapy in veterinary medicine. J Transl Med 2012;10:234. DOI PubMed PMC2317144410.1186/1479-5876-10-234PMC3543347

[26] OmarNB, BentleyRT, CrossmanDK, Safety and interim survival data after intracranial administration of M032, a genetically engineered oncolytic HSV-1 expressing IL-12, in pet dogs with sporadic gliomas. Neurosurg Focus 2021;50:E5. DOI PubMed PMC10.3171/2020.11.FOCUS20844PMC838315533524948

[27] GoulartMR, HlavatySI, ChangYM, Phenotypic and transcriptomic characterization of canine myeloid-derived suppressor cells. Sci Rep 2019;9:3574. DOI PubMed PMC3083760310.1038/s41598-019-40285-3PMC6400936

[28] JacksonK, MilnerRJ, DotyA, Analysis of canine myeloid-derived suppressor cells (MDSCs) utilizing fluorescence-activated cell sorting, RNA protection mediums to yield quality RNA for single-cell RNA sequencing. Vet Immunol Immunopathol 2021;231:110144. DOI PubMed10.1016/j.vetimm.2020.11014433278779

[29] MasonNJ, CoughlinCM, OverleyB, RNA-loaded CD40-activated B cells stimulate antigen-specific T-cell responses in dogs with spontaneous lymphoma. Gene Ther 2008;15:955–65. DOI PubMed1833784110.1038/gt.2008.22

[30] MoreiraML, DornelesEM, SoaresRP, Cross-reactivity of commercially available anti-human monoclonal antibodies with canine cytokines: establishment of a reliable panel to detect the functional profile of peripheral blood lymphocytes by intracytoplasmic staining. Acta Vet Scand 2015;57:51. DOI PubMed PMC2636286010.1186/s13028-015-0142-yPMC4566394

[31] RotheK, BismarckD, BüttnerM, AlberG, von ButtlarH. Canine peripheral blood CD4+CD8+ double-positive Tcell subpopulations exhibit distinct Tcell phenotypes and effector functions. Vet Immunol Immunopathol 2017;185:48–56. DOI PubMed2824200210.1016/j.vetimm.2017.01.005

[32] DaltonMF, StilwellJM, KrimerPM, MillerAD, RissiDR. Clinicopathologic features, diagnosis, and characterization of the immune cell population in canine choroid plexus tumors. Front Vet Sci 2019;6:224. DOI PubMed PMC3138039810.3389/fvets.2019.00224PMC6646530

[33] RissiDR, PorterBF, BoudreauCE, KrimerPM, MillerAD. Immunohistochemical characterization of immune cell infiltration in feline glioma. J Comp Pathol 2018;160:15–22. DOI PubMed2972971710.1016/j.jcpa.2018.02.003

[34] MasonN, BaileyC, PiazzaE, Abstract 1693: Multi-national, multi-center collaboration to develop a novel gene expression tool for comparative translational immuno-oncology. Cancer Res 2021;81:1693. DOI

[35] FraserAR, BacciB, le ChevoirMA, LongSN. Epidermal growth factor receptor and Ki-67 expression in canine gliomas. Vet Pathol 2016;53:1131–7. DOI PubMed2715454210.1177/0300985816644301

[36] Pi CastroD, José-LópezR, Fernández FloresF, Expression of FOXP3 in canine gliomas: immunohistochemical study of tumor-infiltrating regulatory lymphocytes. J Neuropathol Exp Neurol 2020;79:184–93. DOI PubMed3184603810.1093/jnen/nlz120

[37] ToedebuschR, GrodzkiAC, DickinsonPJ, Glioma-associated microglia/macrophages augment tumorigenicity in canine astrocytoma, a naturally occurring model of human glioma. Neurooncol Adv 2021;3:vdab062. DOI PubMed PMC10.1093/noajnl/vdab062PMC819390134131649

[38] FilleyA, HenriquezM, BhowmikT, Immunologic and gene expression profiles of spontaneous canine oligodendrogliomas. J Neurooncol 2018;137:469–79. DOI PubMed PMC2933075010.1007/s11060-018-2753-4PMC5924594

[39] SlomaEA, CrenetiCT, ErbHN, MillerAD. Characterization of inflammatory changes associated with canine oligodendroglioma. J Comp Pathol 2015;153:92–100. DOI PubMed2614572310.1016/j.jcpa.2015.05.003

[40] TomaszewskiW, Sanchez-PerezL, GajewskiTF, SampsonJH. Brain tumor microenvironment and host state: implications for immunotherapy. Clin Cancer Res 2019;25:4202–10. DOI PubMed PMC3080401910.1158/1078-0432.CCR-18-1627PMC6635001

[41] BonaventuraP, ShekarianT, AlcazerV, Cold tumors: a therapeutic challenge for immunotherapy. Front Immunol 2019;10:168. DOI PubMed PMC3080012510.3389/fimmu.2019.00168PMC6376112

[42] LouieRJ, PerezMC, JajjaMR, Real-world outcomes of talimogene laherparepvec therapy: a multi-institutional experience. J Am Coll Surg 2019;228:644–9. DOI PubMed3069007610.1016/j.jamcollsurg.2018.12.027

[43] AndtbackaRHI, CollichioFA, AmatrudaT, OPTiM: a randomized phase III trial of talimogene laherparepvec (T-VEC) versus subcutaneous (SC) granulocyte-macrophage colony-stimulating factor (GM-CSF) for the treatment (tx) of unresected stage IIIB/C and IV melanoma. JCO 2013;31:LBA9008. DOI

[44] FrankeV, BergerDMS, KlopWMC, High response rates for T-VEC in early metastatic melanoma (stage IIIB/C-IVM1a). Int J Cancer 2019;145:974–8. DOI PubMed3069455510.1002/ijc.32172

[45] RibasA, DummerR, PuzanovI, Oncolytic virotherapy promotes intratumoral T cell infiltration and improves anti-PD-1 immunotherapy. Cell 2017;170:1109–19.e10. DOI PubMed PMC2888638110.1016/j.cell.2017.08.027PMC8034392

[46] KaufmanHL, KimDW, DeRaffeleG, MitchamJ, CoffinRS, Kim-SchulzeS. Local and distant immunity induced by intralesional vaccination with an oncolytic herpes virus encoding GM-CSF in patients with stage IIIc and IV melanoma. Ann Surg Oncol 2010;17:718–30. DOI PubMed1991591910.1245/s10434-009-0809-6

[47] BenenciaF, CourrègesMC, Conejo-GarcíaJR, HSV oncolytic therapy upregulates interferon-inducible chemokines and recruits immune effector cells in ovarian cancer. Mol Ther 2005;12:789–802. DOI PubMed1592554410.1016/j.ymthe.2005.03.026

[48] ReinertLS, LopušnáK, WintherH, Sensing of HSV-1 by the cGAS-STING pathway in microglia orchestrates antiviral defence in the CNS. Nat Commun 2016;7:13348. DOI PubMed PMC2783070010.1038/ncomms13348PMC5109551

[49] MaW, HeH, WangH. Oncolytic herpes simplex virus and immunotherapy. BMC Immunol 2018;19:40. DOI PubMed PMC3056346610.1186/s12865-018-0281-9PMC6299639

[50] KaufmanHL, KohlhappFJ, ZlozaA. Oncolytic viruses: a new class of immunotherapy drugs. Nat Rev Drug Discov 2015;14:642–62. DOI PubMed PMC2632354510.1038/nrd4663PMC7097180

[51] NayakL, MolinaroAM, PetersK, Randomized phase II and biomarker study of pembrolizumab plus bevacizumab versus pembrolizumab alone for patients with recurrent glioblastoma. Clin Cancer Res 2021;27:1048–57. DOI PubMed PMC3319949010.1158/1078-0432.CCR-20-2500PMC8284901

[52] IorgulescuJB, GokhalePC, SperanzaMC, Concurrent dexamethasone limits the clinical benefit of immune checkpoint blockade in glioblastoma. Clin Cancer Res 2021;27:276–87. DOI PubMed PMC3323943310.1158/1078-0432.CCR-20-2291PMC8034990

[53] OtvosB, AlbanTJ, GrabowskiMM, Preclinical modeling of surgery and steroid therapy for glioblastoma reveals changes in immunophenotype that are associated with tumor growth and outcome. Clin Cancer Res 2021;27:2038–49. DOI PubMed PMC3354207510.1158/1078-0432.CCR-20-3262PMC8026586

[54] ChongsathidkietP, JacksonC, KoyamaS, Sequestration of T cells in bone marrow in the setting of glioblastoma and other intracranial tumors. Nat Med 2018;24:1459–68. DOI PubMed PMC3010476610.1038/s41591-018-0135-2PMC6129206

[55] FriedmanGK, JohnstonJM, BagAK, Oncolytic HSV-1 G207 immunovirotherapy for pediatric high-grade gliomas. N Engl J Med 2021;384:1613–22. DOI PubMed PMC3383862510.1056/NEJMoa2024947PMC8284840

