# Supplementary material for: Evaluation of immunologic parameters in canine glioma patients treated with an oncolytic herpes virus
Source: J Transl Genet Genom. Author manuscript; Available in PMC 2022 Mar 25. (PMC8955901; doi:10.20517/jtgg.2021.31)
Supplement: supplementary materials [file NIHMS1778534-supplement-supplementary_materials.zip › supplementary materials/jtgg-2021-31-SupplementaryFigure1.pdf]

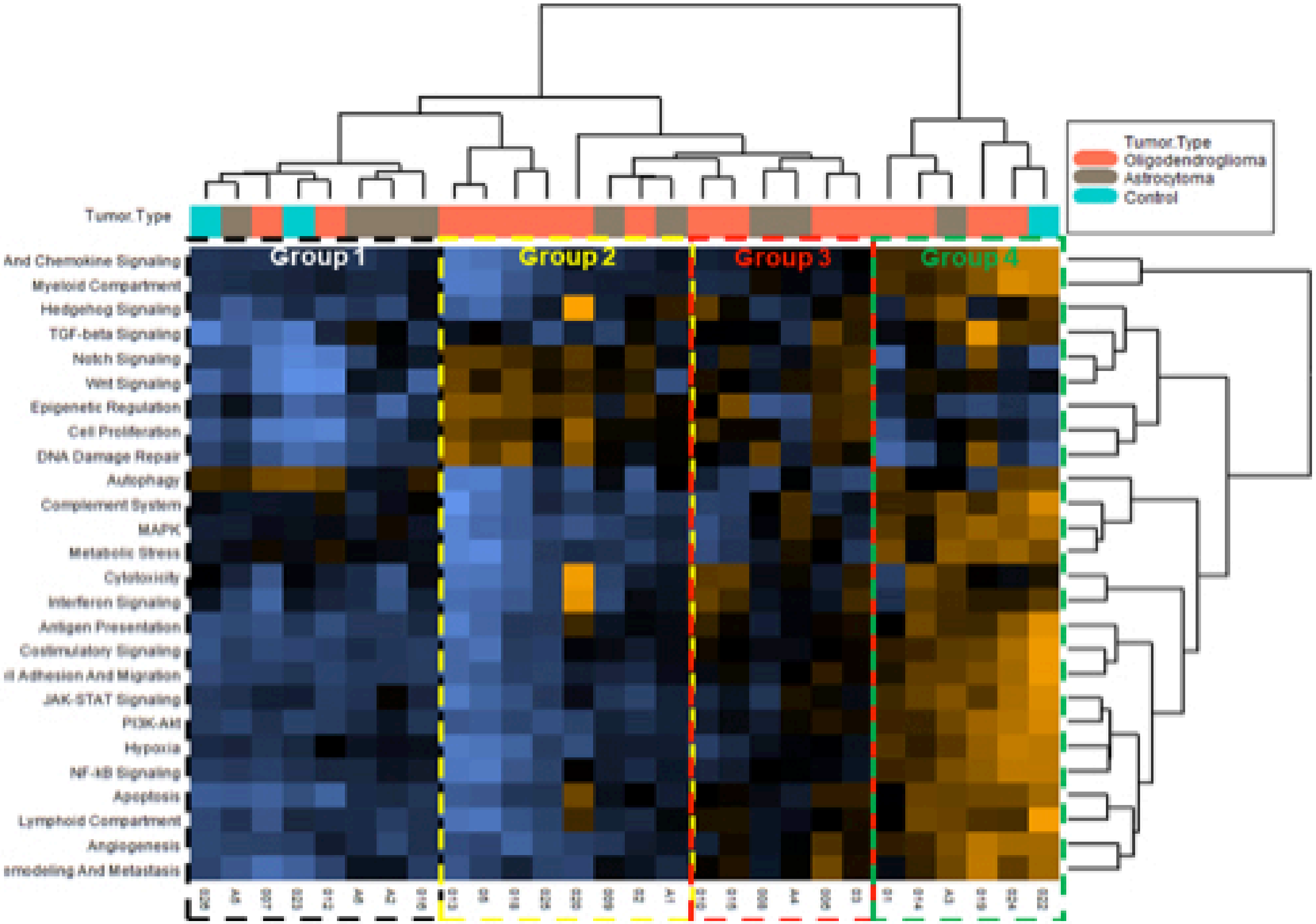

Supplemental Figure 1. Independent clustering of canine glioma tumors based on enrichment for tumor intrinsic or immune related mRNA transcriptional pathways. All samples were analyzed using the advanced analysis module in nSolver (Nanostring Technologies) with patient glioma samples (n = 25; 19 pre-M032 treatment biopsies and 6 non-clinical trial astrocytomas, A1-6) normalized to adjacent normal controls (n = 3). Samples were clustered into 4 groups: (i) Group 1 (n = 8) is enriched in the autophagy pathway, (ii) Group 2 (n = 8) is enriched for cell cycle, proliferation, DNA damage repair, epigenetic regulation, and Notch signaling pathways, (iii) Group 3 (n = 6) displays enrichment for pathways in Group 2 and pathways associated with immune responses, and (iv) Group 4 (n = 5) displays greater level of enrichment for immune pathways.
