# Supplementary material for: Evaluation of immunologic parameters in canine glioma patients treated with an oncolytic herpes virus
Source: J Transl Genet Genom. Author manuscript; Available in PMC 2022 Mar 25. (PMC8955901; doi:10.20517/jtgg.2021.31)
Supplement: supplementary materials [file NIHMS1778534-supplement-supplementary_materials.zip › supplementary materials/jtgg-2021-31-SupplementaryFigure3.pdf]

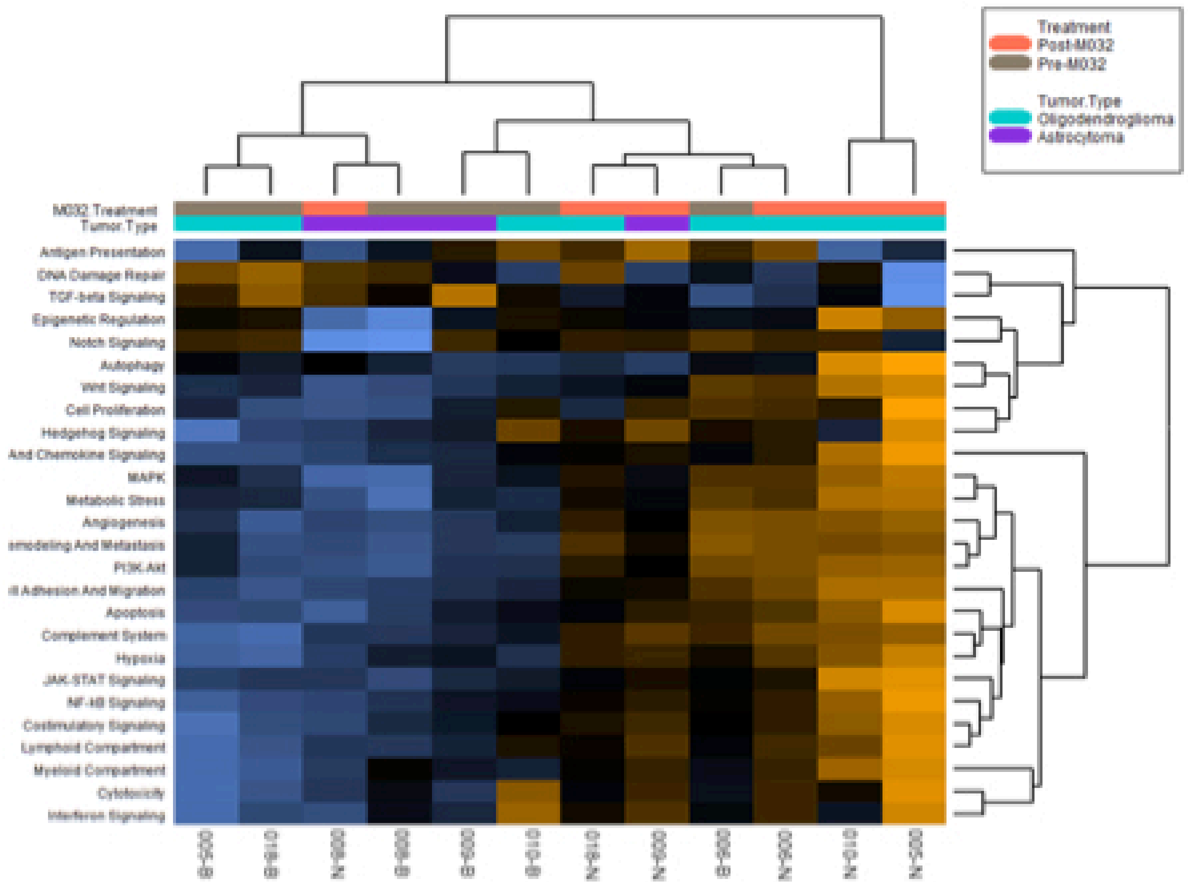

Supplemental Figure 3. Independent clustering of post-M032 treated canine glioma tumors based on enrichment for immune-related and cancer driver pathway signatures likely associated with tumor progression. All samples were analyzed using the advanced analysis module in nSolver (Nanostring Technologies) with matched pre- and post-treatment patient glioma samples (n = 6) including matched samples for four patient oligodendrogliomas (005B, 005N, 006B, 006N, 010B, 010N, 018B and 018N) and two patient astrocytomas (008B, 008N, 009B and 009N). Post-M032 treatment samples were normalized to matched pre-treatment samples. Post-M032 treatment samples (5/6) cluster significantly with immune related mRNA pathways including interferon signaling, cytotoxicity, myeloid and lymphoid compartments, co-stimulatory signaling. In addition, there is increase expression of mRNA driver pathway signatures associated with hypoxia, cell adhesion and migration, PI3K-Akt, remodeling and metastasis, angiogenesis, MAPK, hedgehog signaling, cellular proliferation, and WNT signaling.
