# Supplementary material for: Evaluation of immunologic parameters in canine glioma patients treated with an oncolytic herpes virus
Source: J Transl Genet Genom. Author manuscript; Available in PMC 2022 Mar 25. (PMC8955901; doi:10.20517/jtgg.2021.31)
Supplement: supplementary materials [file NIHMS1778534-supplement-supplementary_materials.zip › supplementary materials/jtgg-2021-31-SupplementaryFigure4.pdf]

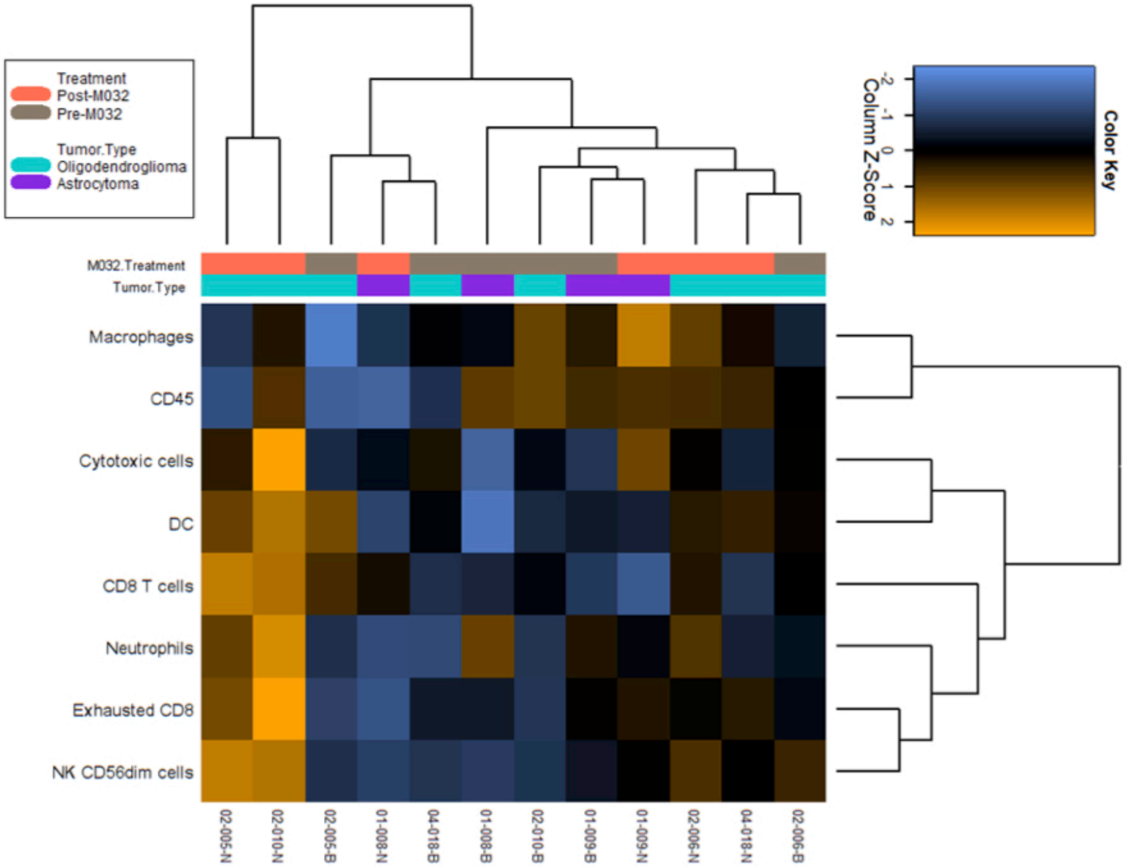

Supplemental Figure 4. M032 treated canine glioma samples exhibit a mixture of immune gene signatures. All samples were analyzed using the advanced analysis module in nSolver (Nanostring Technologies) with matched pre- and post-treatment patient glioma samples (n = 6) including matched samples for four patient oligodendrogliomas (005B, 005N, 006B, 006N, 010B, 010N, 018B and 018N) and two patient astrocytomas (008B, 008N, 009B and 009N). Post-M032 treatment samples were normalized to matched pre-treatment samples. Post-M032 treatment samples exhibit variable clustering with some samples (005N and 010N) exhibiting mRNA gene signatures for NK cells, CD8+ T cells, and dendritic cells, whereas other populations (006N, 009B, 009N, 010B, 018N) exhibit mRNA gene signatures for macrophage and exhausted CD8+ T cells.
