# Supplementary material for: Evaluation of immunologic parameters in canine glioma patients treated with an oncolytic herpes virus
Source: J Transl Genet Genom. Author manuscript; Available in PMC 2022 Mar 25. (PMC8955901; doi:10.20517/jtgg.2021.31)
Supplement: supplementary materials [file NIHMS1778534-supplement-supplementary_materials.zip › supplementary materials/jtgg-2021-31-SupplementaryFigure5.pdf]

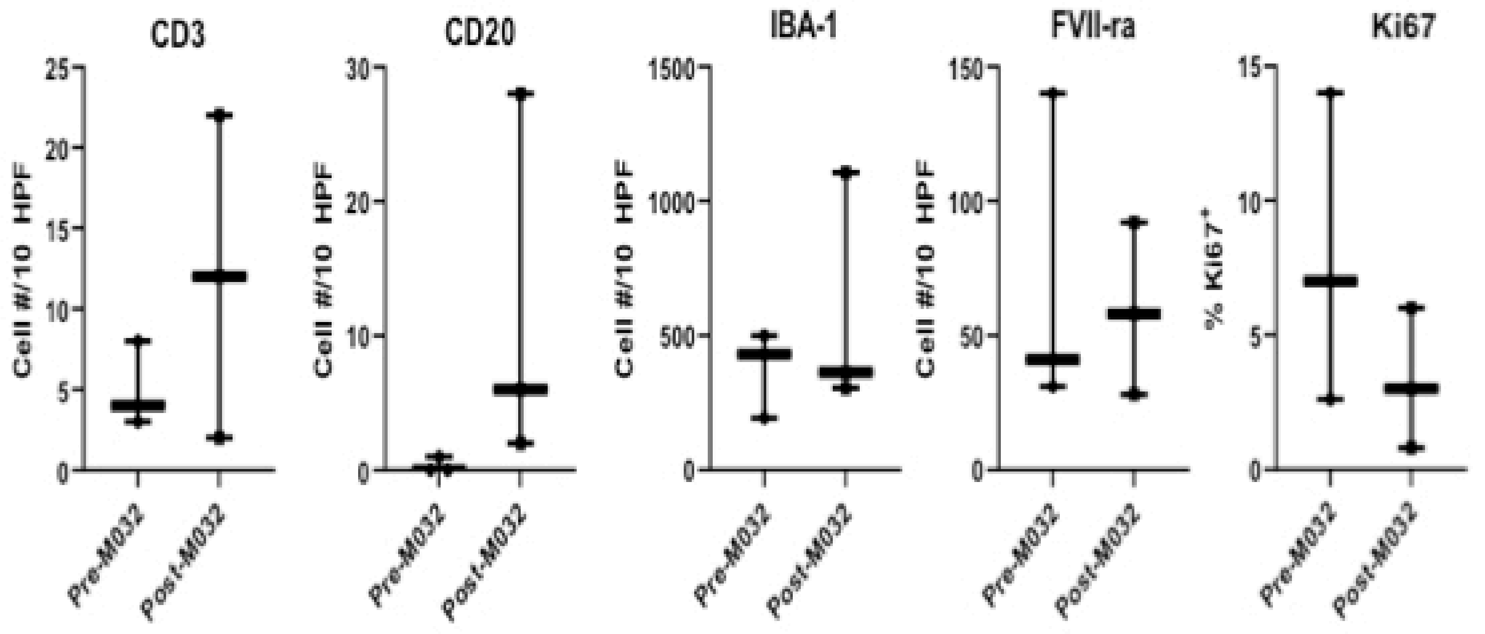

Supplemental Figure 5. Characterization of changes in immune infiltrate and tumor biology post-M032 treatment. A total of three matched pre- and post-M032 treatment samples (005, 008, and 009) were evaluated for $CD3^{+}$ T cell, $CD20^{+}$ B cell, IBA-1^{+} microglial/ macrophage, FVIII-ra^{+} endothelial, and %$Ki67^{+}$ tumor cells to determine if any significant changes were present after M032 treatment. No statistically significant differences were noted in this small sample size.
