# Supplementary material for: Evaluation of immunologic parameters in canine glioma patients treated with an oncolytic herpes virus
Source: J Transl Genet Genom. Author manuscript; Available in PMC 2022 Mar 25. (PMC8955901; doi:10.20517/jtgg.2021.31)
Supplement: supplementary materials [file NIHMS1778534-supplement-supplementary_materials.zip › supplementary materials/jtgg-2021-31-SupplementaryFigure7.pdf]

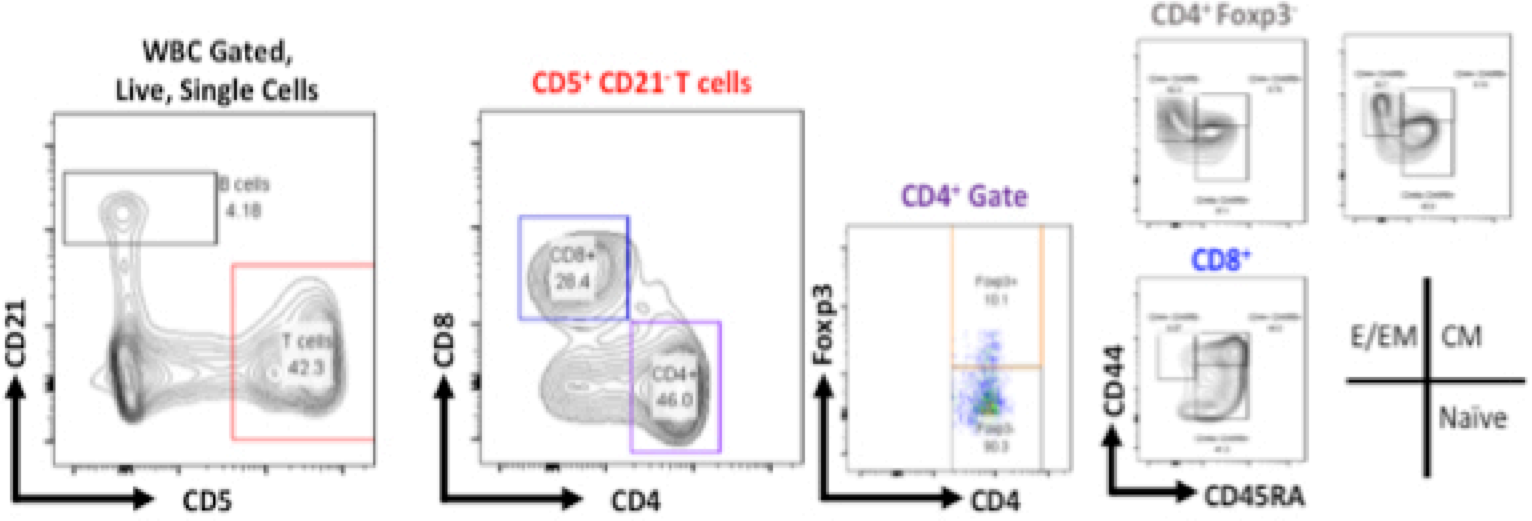

Supplemental Figure 7. Gating strategy for T lymphocytes. T lymphocytes are identified by CD5 expression then subsetted into CD4+ and CD8+ T cells. Regulatory T lymphocytes are identified by intranuclear Foxp3 expression. Activation status of CD4+ and CD8+ is determined by expression of CD44, CD45RA, and CCR7 where CD44hi CD45RA- CCR7- identify effector and effector memory T lymphocytes, CD44hi CD45RA+ CCR7+ identify central memory T lymphocytes, and CD44lo CD45RA+ CCR7+ identify naïve T lymphocytes.
