# Supplementary material for: Evaluation of immunologic parameters in canine glioma patients treated with an oncolytic herpes virus
Source: J Transl Genet Genom. Author manuscript; Available in PMC 2022 Mar 25. (PMC8955901; doi:10.20517/jtgg.2021.31)
Supplement: supplementary materials [file NIHMS1778534-supplement-supplementary_materials.zip › supplementary materials/jtgg-2021-31-SupplementaryFigure8.pdf]

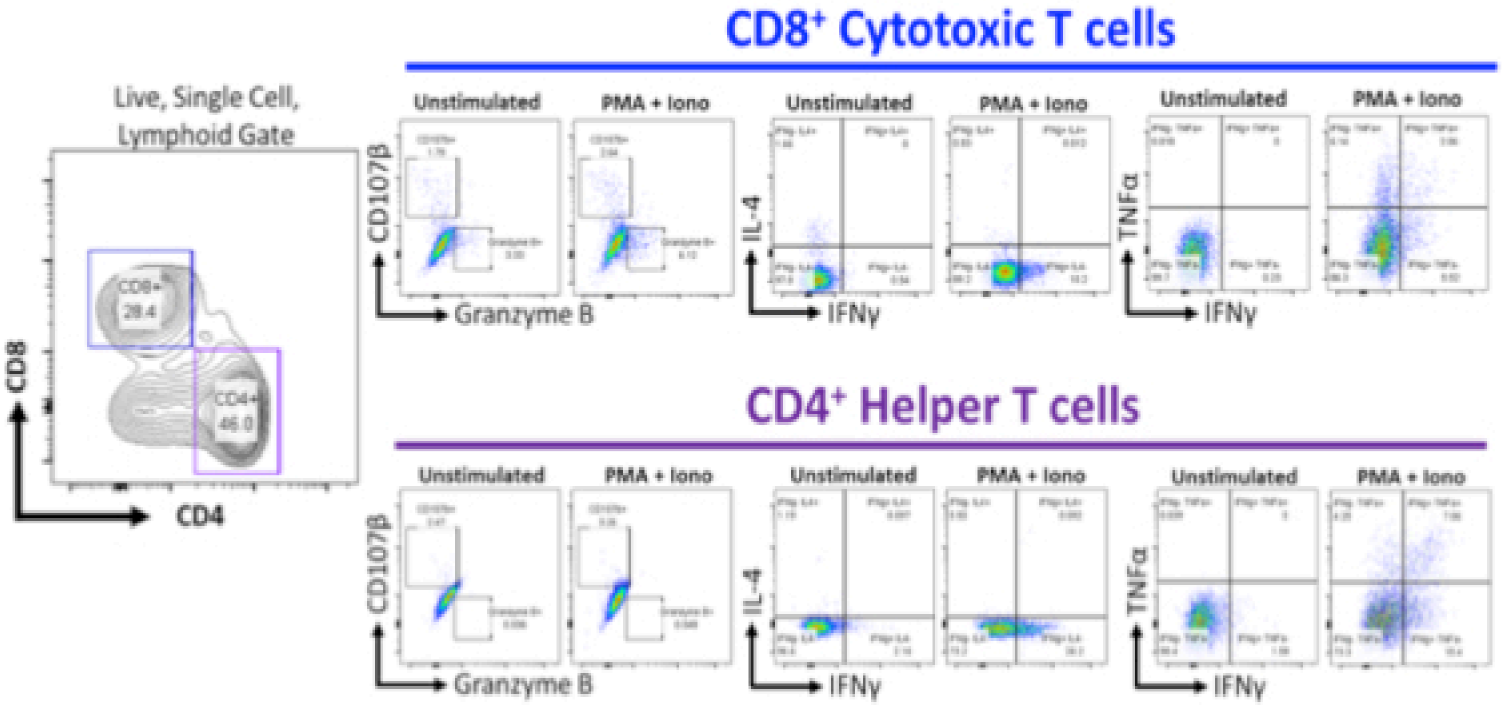

Supplemental Figure 8. Intracellular cytokine staining. Cells were stimulated (10^6 cells/mL) with PMA and ionomycin in the presence of monensin (Invitrogen Leukocyte activation cocktail) for 4 hours then T cell phenotype and function was evaluated by flow cytometry were T cells are identified by CD5 expression then subsetted into CD4⁺ and CD8⁺ T cells. Expression of CD107b (degranulation marker), IFNγ, IL-4, TNFα, and Granzyme B were evaluated by intracytoplasmic staining. Cytoplasmic expression was compared between PMA/ionomycin and unstimulated specimens to determine fold induction of cytokine, CD107b and Granzyme B expression.
