# Supplementary material for: Evaluation of immunologic parameters in canine glioma patients treated with an oncolytic herpes virus
Source: J Transl Genet Genom. Author manuscript; Available in PMC 2022 Mar 25. (PMC8955901; doi:10.20517/jtgg.2021.31)
Supplement: supplementary materials [file NIHMS1778534-supplement-supplementary_materials.zip › supplementary materials/jtgg-2021-31-SupplementaryTable1.pdf]

Supplemental Table 1: CANINE Trial Stage 1 Interim Safety and Survival Data

TABLE 4. Interim data on canine characteristics, viral dosing, tumor pathology, and survival after administration of M032

ID No.	Age at Treatment (yrs)	Weight at Treatment (kg)	Sex-Neuter, Spay, or Intact	Breed	Tumor Location	Prior Treatment	M032 Dose (pfu)	Tumor Pathology	Tumor Grade	Posttreatment Survival (days)
001	6	14.1	M-N	English Bulldog	Lt frontal/parietal lobe	No	1E+06	Oligodendroglioma	Low	108
002	10	24.1	M-N	Boxer	Rt frontal lobe	No	1E+06	Oligodendroglioma	High	372
003	7	23.0	M-N	English Bulldog	Lt parietal/occipital lobe	No	1E+06	Oligodendroglioma	High	232
004	9	7.03	M-N	Yorkshire Terrier	Rt frontal lobe	Yes	1E+07	Astrocytoma	Low	188
005	7	53.0	M-N	Mixed breed	Lt piriform lobe	Yes	1E+07	Oligodendroglioma	High	151
006	6	10.8	M-N	Boston Terrier	Rt forebrain	No	1E+07	Astrocytoma	Undetermined	415
007	7	12.7	M-N	Mixed breed	Rt rostral fossa	No	1E+08	Oligodendroglioma	Undetermined	Left study to pursue alternate treatment
008	6	55.2	F-S	German Shepherd	Rt frontal lobe	No	1E+08	Oligodendroglioma	High	41
009	11	8.6	F-S	Boston Terrier	Rt frontal/parietal lobe	No	1E+08	Astrocytoma	High	11
010	7	34.4	M-N	American Bulldog	Rt temporal lobe	No	1E+09	Oligodendroglioma	High	43
011	4	52.0	M-N	Great Dane	Lt forebrain	No	1E+09	Oligodendroglioma	High	219
012	11.5	11.4	M-N	Boston Terrier	Lt frontal lobe	No	1E+09	Oligodendroglioma	Undetermined	378*
013	8	39.6	F-S	Mixed breed	Rt temporal lobe	No	1E+09	Oligodendroglioma	High	43
014	8	11.6	M-N	Boston Terrier	Lt frontal lobe	No	1E+09	Oligodendroglioma	Low	369*
015	14.5	41.0	M-N	Boxer	Rt temporal lobe	No	N/A	Oligodendroglioma	Low	Died of pneumonia prior to inoculation
016	10	24.1	F-S	Border Collie	Rt forebrain	No	1E+09	Undefined with astrocytic features	Low	362*
017	11	8.47	M-N	Boston Terrier	Rt temporal lobe	No	1E+09	Oligodendroglioma	Low	28
018	9.5	11.6	M-N	French Bulldog	Lt parietal lobe	No	1E+09	Oligodendroglioma	High	70
019	9	5.0	M-N	Yorkshire Terrier	Rt temporal lobe	No	1E+09	Glioblastoma	High	55
020	5	26.0	M-N	Mixed breed	Rt temporal lobe	No	1E+09	Oligodendroglioma	High	28
021	5	28.6	M-N	Golden Retriever	Rt occipital lobe	No	1E+09	Astrocytoma	High	103*
022	6.5	34	M-N	Boxer	Lt temporal lobe	No	N/A	Granulomatous meningoencephalitis	N/A	Died of pneumonia prior to inoculation
023	7	29.1	F-S	Mixed breed	Rt temporal lobe	No	1E+09	Undetermined	Undetermined	39*
024	7.5	33.1	M-I	Boxer	Lt frontal lobe	No	N/A	Glioblastoma	High	Died of pneumonia prior to inoculation
025	6.5	37.2	M-I	Cane Corso	Lt frontal/parietal lobe	No	1E+09	Astrocytoma	High	33*

I = intact; N = neutered; S = spayed.
*** Canines still living.**
