# Supplementary material for: Evaluation of immunologic parameters in canine glioma patients treated with an oncolytic herpes virus
Source: J Transl Genet Genom. Author manuscript; Available in PMC 2022 Mar 25. (PMC8955901; doi:10.20517/jtgg.2021.31)
Supplement: supplementary materials [file jtgg-2021-31-SupplementaryFigure2.pdf › supplementary materials/jtgg-2021-31-SupplementaryFigure2.pdf]

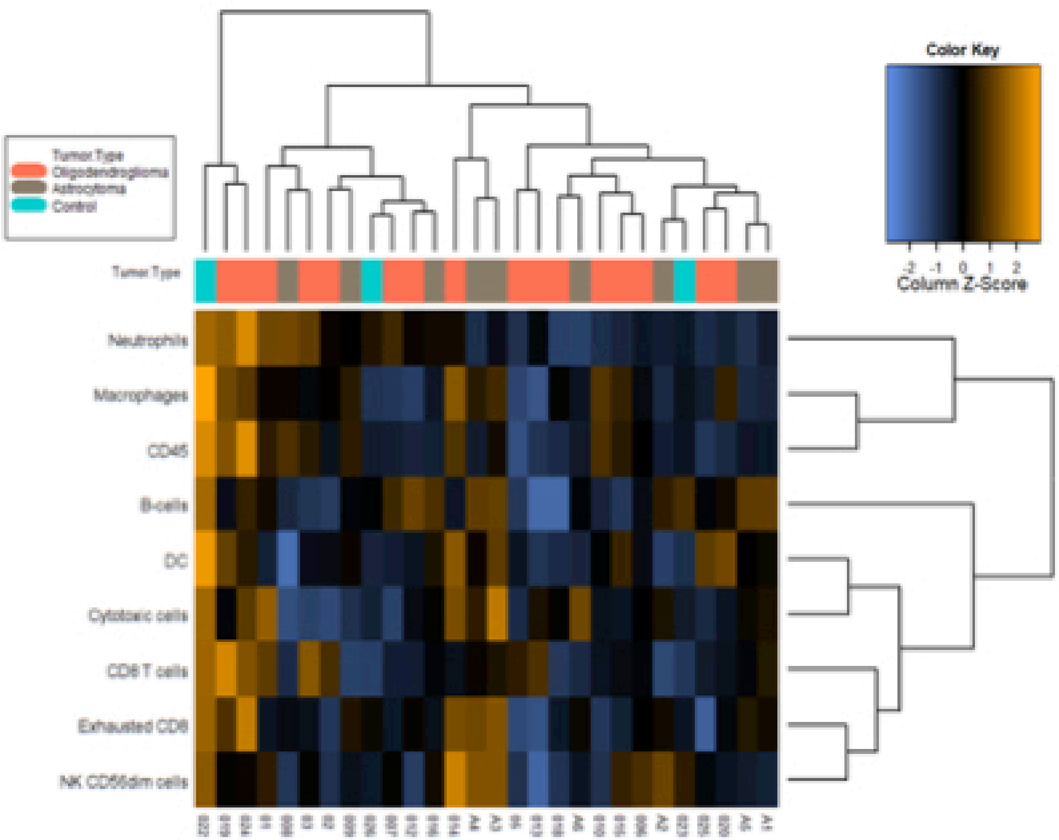

Supplemental Figure 2. Independent clustering of canine glioma tumor subtypes based on enrichment for immune subsets. All samples were analyzed using the advanced analysis module in nSolver (Nanostring Technologies) with patient glioma samples (n = 22) normalized to adjacent normal controls (n = 3). Samples cluster to a lesser degree into those expressing mRNA gene signatures associated with an array of immune infiltrate on the left side of the heat map versus those on the right exhibiting fewer immune cell mRNA gene signatures.
