# Supplementary material for: Evaluation of immunologic parameters in canine glioma patients treated with an oncolytic herpes virus
Source: J Transl Genet Genom. Author manuscript; Available in PMC 2022 Mar 25. (PMC8955901; doi:10.20517/jtgg.2021.31)
Supplement: supplementary materials [file jtgg-2021-31-SupplementaryFigure6.pdf › supplementary materials/jtgg-2021-31-SupplementaryFigure6.pdf]

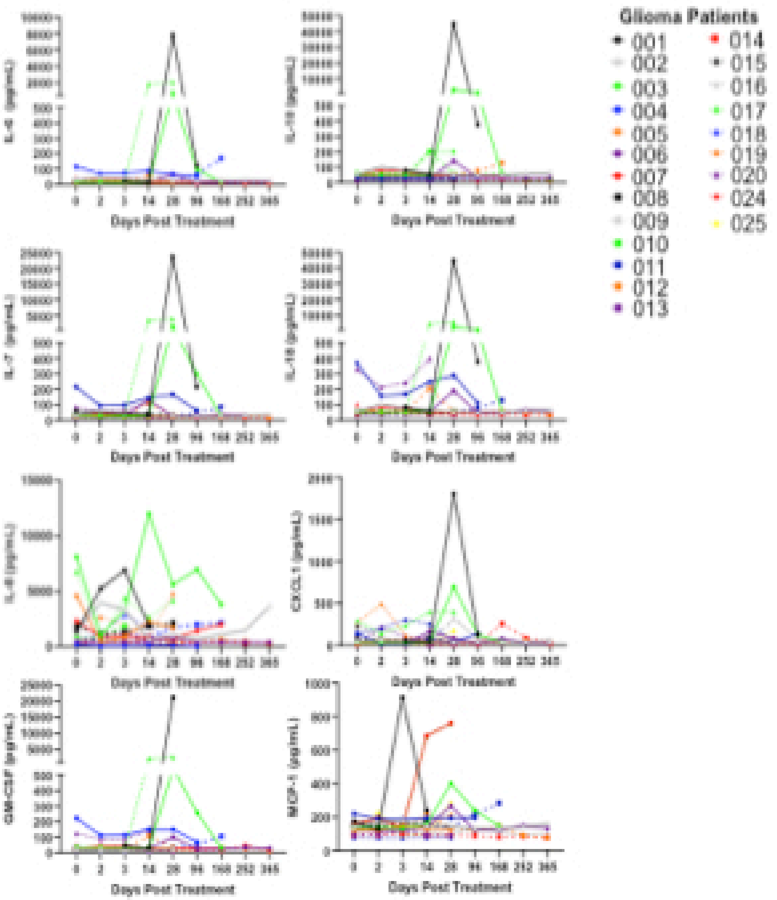

Supplemental Figure 6. Analysis of peripheral blood for correlates of tumor immune responses. Immune function was characterized using multianalyte ELISA to determine cytokine and chemokine expression in peripheral blood and plasma obtained at specific time points after M032 treatment.
