# Supplementary material for: Evaluation of immunologic parameters in canine glioma patients treated with an oncolytic herpes virus
Source: J Transl Genet Genom. Author manuscript; Available in PMC 2022 Mar 25. (PMC8955901; doi:10.20517/jtgg.2021.31)
Supplement: supplementary materials [file NIHMS1778534-supplement-supplementary_materials.zip › supplementary materials/jtgg-2021-31-SupplementaryTable4.pdf]

Supplemental Table 4: Top 20 Differentially expressed mRNA genes in M032 treated vs. treatment naïve canine glioma patients

mRNA	Linear Fold Change	P value	Function
C1QBP	3	7.74×10^{-5}	Complement pathway
IFNA7	15.8	0.00128	Cytokine, Interferon signaling
IL1A	8.04	0.000292	Cytokine
AICDA	5.56	0.000645	T and B cell function
TNFRSF12	13.2	0.00245	Angiogenesis
KLRA1	5.66	0.00334	Cytotoxicity
CXCR5	4.7	0.00366	B cell function
NT5E	7.92	0.00485	Transporter, immune suppression
IL29L	6.07	0.00582	Cytokine, Interferon signaling
SELE	4.82	0.0122	Cell adhesion, migration
C6	4.66	0.0169	Complement pathway
CD59	3.35	0.0178	Complement pathway, immune suppression

TNFRSF11B	7.1	0.0183	Cytokine signaling, TNF superfamily
PDCD1 (PD-1)	4.97	0.02	Co-stimulation inhibitor
SAA1	11.9	0.0209	Cytokine/chemokine signaling, acute inflammation
CD276 (B7-H3)	1.95	0.0274	Co-stimulation inhibitor
PVR	2.13	0.0288	Co-stimulation inhibitor
TLR9	2.79	0.0325	Toll-like receptor
IL-16	7.26	0.0334	Cytokine

Differential gene expression in canine glioma tumor tissue post-M032 treatment (n = 3) compared to patient matched treatment naïve canine glioma samples (n = 3). The focus is on genes associated with innate and adaptive immune responses.
