# Supplementary material for: Evaluation of immunologic parameters in canine glioma patients treated with an oncolytic herpes virus
Source: J Transl Genet Genom. Author manuscript; Available in PMC 2022 Mar 25. (PMC8955901; doi:10.20517/jtgg.2021.31)
Supplement: supplementary materials [file NIHMS1778534-supplement-supplementary_materials.zip › supplementary materials/jtgg-2021-31-SupplementaryTable2.pdf]

Supplemental Table 2: Nanostring Canine IO Panel
(115000465: Canine IO CSO+PS, XT-CSPS-CIO-12; 100052: Master Kit, NAA-AKIT-012)

Gene	Canine IO	
ABCB1	+	
ABCF1	+	
ABL1	+	
ACOD1	+	
ACVR1	+	
ADA	+	
ADAMTS2	+	
ADGRE5	+	
ADORA2A	+	
ADORA3	+	
AICDA	+	
AIRE	+	
AKT1	+	
AKT3	+	
ALCAM	+	
AMBP	+	
ANKRD22	+	
ANXA1	+	
APC	+	
APOE	+	
APP	+	
AR	+	
ARG1	+	
ARG2	+	
ATF1	+	
ATF2	+	
ATG10	+	
ATG12	+	
ATG16L1	+	
ATG5	+	
ATG7	+	
ATM	+	
ATRX	+	
AXL	+	
B2M	+	
BATF	+	
BAX	+	
BCL10	+	
BCL2	+	
BCL2L1	+	
BCL6	+	
BCR	+	
BHLHE40	+	
BID	+	

BIRC5	+	
BLK	+	
BLNK	+	
BMI1	+	
BRAF	+	
BRCA1	+	
BRCA2	+	
BST1	+	
BST2	+	
BTK	+	
BTLA	+	
C1QA	+	
C1QB	+	
C1QBP	+	
C1R	+	
C1S	+	
C2	+	
C3	+	
C3AR1	+	
C4BPA	+	
C5	+	
C6	+	
C7	+	
C8A	+	
C8B	+	
C8G	+	
C9	+	
CALHM6	+	
CAMP	+	
CARD11	+	
CARD9	+	
CASP10	+	
CASP3	+	
CASP8	+	
CCL1	+	
CCL13	+	
CCL14	+	
CCL16	+	
CCL17	+	
CCL19	+	
CCL2	+	
CCL20	+	
CCL21	+	
CCL22	+	
CCL23	+	
CCL24	+	

CCL25	+	
CCL26	+	
CCL27	+	
CCL28	+	
CCL3	+	
CCL4	+	
CCL5	+	
CCL7	+	
CCL8	+	
CCND1	+	
CCND2	+	
CCND3	+	
CCR1	+	
CCR10	+	
CCR2	+	
CCR3	+	
CCR4	+	
CCR5	+	
CCR6	+	
CCR7	+	
CCR9	+	
CCRL2	+	
CD14	+	
CD160	+	
CD163	+	
CD164	+	
CD180	+	
CD19	+	
CD1A6	+	
CD1B	+	
CD1C	+	
CD1D	+	
CD1E	+	
CD2	+	
CD200	+	
CD207	+	
CD209	+	
CD22	+	
CD244	+	
CD247	+	
CD27	+	
CD274	+	
CD276	+	
CD28	+	
CD34	+	
CD36	+	

CD37	+	
CD38	+	
CD3D	+	
CD3E	+	
CD3EAP	+	
CD3G	+	
CD4	+	
CD40	+	
CD40LG	+	
CD44	+	
CD47	+	
CD48	+	
CD5	+	
CD53	+	
CD55	+	
CD58	+	
CD59	+	
CD6	+	
CD63	+	
CD68	+	
CD7	+	
CD70	+	
CD74	+	
CD79A	+	
CD79B	+	
CD80	+	
CD81	+	
CD83	+	
CD84	+	
CD86	+	
CD8A	+	
CD8B	+	
CD9	+	
CD96	+	
CD99	+	
CDH1	+	
CDH5	+	
CDK1	+	
CDK4	+	
CDKN1A	+	
CDKN2A	+	
CDKN2B	+	
CDKN2C	+	
CEACAM1	+	
CEBPA	+	
CEBPB	+	

CFB	+	
CFD	+	
CFI	+	
CFP	+	
CH25H	+	
CHEK2	+	
CHUK	+	
CIITA	+	
CISH	+	
CKLF	+	
CLDN1	+	
CLEC4A	+	
CLEC5A	+	
CLEC7A	+	
CLU	+	
CMA1	+	
CMKLR1	+	
CNP	+	
COL1A1	+	
COL3A1	+	
COLEC12	+	
CPA3	+	
CR1L	+	
CR2	+	
CREB1	+	
CREB5	+	
CREBBP	+	
CRP	+	
CSF1	+	
CSF1R	+	
CSF2	+	
CSF2RB	+	
CSF3	+	
CSF3R	+	
CTLA4	+	
CTSG	+	
CTSH	+	
CTSS	+	
CTSW	+	
CX3CL1	+	
CX3CR1	+	
CXCL10	+	
CXCL11	+	
CXCL12	+	
CXCL13	+	

CXCL14	+	
CXCL16	+	
CXCL8	+	
CXCR1	+	
CXCR2	+	
CXCR3	+	
CXCR4	+	
CXCR5	+	
CXCR6	+	
CYBB	+	
CYFIP2	+	
CYLD	+	
DDR1	+	
DDX5	+	
DDX58	+	
DEFB1	+	
DLA-12	+	
DLA-64	+	
DLA-79	+	
DLA-DMA	+	
DLA-DMB	+	
DLA-DOB	+	
DLA-DQA1	+	
DLA-DQB1	+	
DLA-DRA	+	
DLA88	+	
DMBT1	+	
DNAJC14	+	
DNMT3A	+	
DOCK2	+	
DOK1	+	
DOK2	+	
DUSP1	+	
DUSP6	+	
EBI3	+	
EGFR	+	
EGR1	+	
ELANE	+	
ENTPD1	+	
EOMES	+	
EP300	+	
EPCAM	+	
EPHA5	+	

EPSTI1	+	
ERBB2	+	
ERBB3	+	
ERBB4	+	
ERCC3	+	
ESR1	+	
ESR2	+	
ETS1	+	
F13A1	+	
FABP7	+	
FADD	+	
FAS	+	
FASLG	+	
FAT1	+	
FBXW11	+	
FCAR	+	
FCER1A	+	
FCER1G	+	
FCER2	+	
FCGR1A	+	
FCRL2	+	
FGFR1	+	
FGFR3	+	
FLT3	+	
FLT3LG	+	
FN1	+	
FOS	+	
FOXA1	+	
FOXP3	+	
FPR2	+	
FYN	+	
G6PD	+	
GATA3	+	
GBP5	+	
GFAP	+	
GH1	+	
GPI	+	
GSK3A	+	
GSK3B	+	
GUSB	+	
GZMA	+	
GZMB	+	
GZMH	+	

GZMK	+	
HAMP	+	
HAVCR2	+	
HBEGF	+	
HCK	+	
HCST	+	
HDC	+	
HIF1A	+	
HLA-DRB1	+	
HMGB1	+	
HMGCR	+	
HNF1A	+	
HRAS	+	
HSD11B1	+	
ICAM1	+	
ICAM2	+	
ICAM3	+	
ICAM4	+	
ICOS	+	
ICOSLG	+	
IDH1	+	
IDH2	+	
IDO1	+	
IDO2	+	
IFGGC1	+	
IFI35	+	
IFIH1	+	
IFIT2	+	
IFNA7	+	
IFNAR1	+	
IFNAR2	+	
IFNB1	+	
IFNG	+	
IFNGR1	+	
IGF1R	+	
IGF2R	+	
IGHG	+	
IGHM	+	
IKBKB	+	
IKBKE	+	
IKBKG	+	
IL10	+	
IL10RA	+	
IL11	+	
IL11RA	+	
IL12A	+	

IL12B	+	
IL12RB1	+	
IL12RB2	+	
IL13	+	
IL13RA1	+	
IL13RA2	+	
IL15	+	
IL15RA	+	
IL16	+	
IL17A	+	
IL17B	+	
IL17F	+	
IL17RA	+	
IL17RB	+	
IL18	+	
IL18R1	+	
IL18RAP	+	
IL19	+	
IL1A	+	
IL1B	+	
IL1R1	+	
IL1R2	+	
IL1RAP	+	
IL1RL1	+	
IL1RL2	+	
IL1RN	+	
IL2	+	
IL21	+	
IL21R	+	
IL22	+	
IL22RA1	+	
IL22RA2	+	
IL23A	+	
IL23R	+	
IL24	+	
IL25	+	
IL26	+	
IL27	+	
IL29L	+	
IL2RA	+	
IL2RB	+	
IL2RG	+	
IL3	+	
IL31RA	+	
IL32	+	
IL34	+	

IL3RA	+	
IL4	+	
IL4R	+	
IL5	+	
IL5RA	+	
IL6	+	
IL6R	+	
IL6ST	+	
IL7	+	
IL7R	+	
IL9	+	
ILF3	+	
INPPL1	+	
IRAK1	+	
IRAK2	+	
IRAK4	+	
IRF1	+	
IRF2	+	
IRF3	+	
IRF4	+	
IRF5	+	
IRF8	+	
ISG15	+	
ISG20	+	
ITGA1	+	
ITGA2	+	
ITGA2B	+	
ITGA4	+	
ITGA5	+	
ITGA6	+	
ITGAE	+	
ITGAL	+	
ITGAM	+	
ITGAV	+	
ITGAX	+	
ITGB1	+	
ITGB2	+	
ITGB3	+	
ITGB4	+	
ITK	+	
JAK1	+	
JAK2	+	
JAK3	+	
JAM3	+	
JAML	+	

KDM5C	+	
KDR	+	
KIT	+	
KLRA1	+	
KLRB1	+	
KLRD1	+	
KLRF1	+	
KLRG1	+	
KLRK1	+	
KMT2A	+	
KMT2D	+	
KRT14	+	
KRT18	+	
KRT7	+	
KRT8	+	
LAG3	+	
LAIR1	+	
LAMP1	+	
LAMP2	+	
LAP3	+	
LBP	+	
LCK	+	
LCN2	+	
LCP1	+	
LFNG	+	
LGALS1	+	
LGALS3	+	
LIF	+	
LOC100049	+	
LOC100683	+	
LOC100683	+	
LOC100686	+	
LOC100856	+	
LOC102153	+	
LOC102154	+	
LOC102155	+	
LOC102156	+	
LOC102156	+	
LOC102156	+	
LOC102156	+	
LOC106557	+	
LOC111094	+	
LOC475935	+	
LOC476396	+	

LOC476900	+	
LOC477699	+	
LOC478984	+	
LOC480600	+	
LOC481722	+	
LOC483397	+	
LOC484306	+	
LOC484343	+	
LOC487977	+	
LOC488947	+	
LOC490356	+	
LOC606890	+	
LOC609023	+	
LOC612539	+	
LRP1	+	
LTA	+	
LTB	+	
LTBR	+	
LTF	+	
LTK	+	
LY86	+	
LY9	+	
LY96	+	
LYN	+	
MAF	+	
MAP2	+	
MAP2K1	+	
MAP2K2	+	
MAP2K4	+	
MAP3K1	+	
MAP3K5	+	
MAP3K7	+	
MAP4K2	+	
MAPK1	+	
MAPK11	+	
MAPK14	+	
MAPK3	+	
MAPK8	+	
MAPKAPK2	+	
MARCO	+	
MASP1	+	
MASP2	+	
MAVS	+	
MBL2	+	
MCAM	+	
MDM4	+	

MEF2C	+	
MEFV	+	
MERTK	+	
MET	+	
MGMT	+	
MIF	+	
MKI67	+	
MME	+	
MMP1	+	
MMP9	+	
MR1	+	
MRC1	+	
MRPL19	+	
MS4A1	+	
MS4A2	+	
MSR1	+	
MST1R	+	
MTOR	+	
MUC1	+	
MX1	+	
MYC	+	
MYD88	+	
NCAM1	+	
NCF4	+	
NCR1	+	
NCR3	+	
NDRG1	+	
NF1	+	
NFATC1	+	
NFATC2	+	
NFATC3	+	
NFATC4	+	
NFKB1	+	
NFKB2	+	
NFKBIA	+	
NINJ2	+	
NKG7	+	
NLRC5	+	
NLRP3	+	
NOD1	+	
NOD2	+	
NOS2	+	
NOTCH1	+	
NPNT	+	
NR1H3	+	

NRAS	+	
NRDE2	+	
NRP1	+	
NT5E	+	
NTRK1	+	
NUP62	+	
OAS2	+	
OAS3	+	
OAZ1	+	
OLIG2	+	
OSM	+	
PALB2	+	
PARP1	+	
PAX5	+	
PCNA	+	
PDCD1	+	
PDCD1LG2	+	
PDGFC	+	
PDGFRA	+	
PDGFRB	+	
PDPN	+	
PECAM1	+	
PGR	+	
PIK3C2B	+	
PIK3C2G	+	
PIK3CA	+	
PIK3CD	+	
PIK3CG	+	
PIK3R1	+	
PIN1	+	
PIP	+	
PLA2G1B	+	
PLAU	+	
PLAUR	+	
PLCG1	+	
PNMA1	+	
PNOC	+	
POLR2A	+	
POU2AF1	+	
POU2F2	+	
PPARG	+	
PPARGC1A	+	
PPBP	+	

PRDM1	+	
PRF1	+	
PRKCD	+	
PRKCE	+	
PSMB10	+	
PSMB8	+	
PSMB9	+	
PSMC4	+	
PTEN	+	
PTGDR2	+	
PTGER2	+	
PTGER4	+	
PTGS2	+	
PTHLH	+	
PTK2	+	
PTPRC	+	
PUM1	+	
PVR	+	
PYCARD	+	
RAD51	+	
RAF1	+	
RAG2	+	
RB1	+	
REL	+	
RELA	+	
RELB	+	
RET	+	
RIPK2	+	
RORA	+	
RORC	+	
RPS6	+	
RUNX1	+	
S100A10	+	
S100A12	+	
S100A4	+	
S100A8	+	
S100A9	+	
S100B	+	
SAA1	+	
SDC4	+	
SDHA	+	
SELE	+	
SELL	+	
SELPLG	+	
SERPINB2	+	

SERPING1	+	
SETD2	+	
SF3A1	+	
SH2D1A	+	
SHMT2	+	
SIGIRR	+	
SIGLEC1	+	
SLAMF1	+	
SLAMF6	+	
SLAMF7	+	
SLC11A1	+	
SLC16A1	+	
SLC16A3	+	
SMAD2	+	
SMAD3	+	
SMARCA4	+	
SOCS1	+	
SOX10	+	
SPIB	+	
SPP1	+	
SPRY2	+	
SRC	+	
ST6GAL1	+	
STAT1	+	
STAT2	+	
STAT3	+	
STAT4	+	
STAT5A	+	
STAT5B	+	
STAT6	+	
STK11	+	
STK11IP	+	
SYK	+	
TAL1	+	
TANK	+	
TAP1	+	
TAPBP	+	
TBC1D10B	+	
TBK1	+	
TBP	+	
TBX21	+	
TCF7	+	
TERT	+	

TFE3	+	
TFEB	+	
TFRC	+	
TGFB1	+	
TGFB2	+	
THBD	+	
THBS1	+	
THY1	+	
TICAM1	+	
TICAM2	+	
TIGIT	+	
TIRAP	+	
TLK2	+	
TLR1	+	
TLR10	+	
TLR2	+	
TLR3	+	
TLR4	+	
TLR5	+	
TLR6	+	
TLR7	+	
TLR8	+	
TLR9	+	
TMUB2	+	
TNF	+	
TNFAIP3	+	
TNFRSF11A	+	
TNFRSF11B	+	
TNFRSF12A	+	
TNFRSF13B	+	
TNFRSF13C	+	
TNFRSF14	+	
TNFRSF17	+	
TNFRSF18	+	
TNFRSF1A	+	
TNFRSF1B	+	
TNFRSF4	+	
TNFRSF8	+	
TNFRSF9	+	
TNFSF10	+	
TNFSF11	+	
TNFSF12	+	
TNFSF13	+	
TNFSF13B	+	
TNFSF14	+	
TNFSF15	+	

Text

TNFSF18	+	
TNFSF4	+	
TNFSF8	+	
TNKS	+	
TNKS2	+	
TOLLIP	+	
TOX	+	
TP53	+	
TP63	+	
TRAC	+	
TRAF1	+	
TRAF2	+	
TRAF3	+	
TRAF6	+	
TRAT1	+	
TRBC	+	
TREM1	+	
TREM2	+	
TRGC2	+	
TRGC3	+	
TRGC8	+	
TRIB1	+	
TSC2	+	
TUBB3	+	
TXK	+	
TXNIP	+	
TYK2	+	
TYMS	+	
TYROBP	+	
UBB	+	
VCAM1	+	
VEGFA	+	
VEGFC	+	
VIM	+	
VSIR	+	
XCR1	+	
ZAP70	+	
ZEB1	+	

The Canine IO panel was developed specifically for use with paraffin-embedded canine tumor tissue from canine cancer patients through a collaboration among members of the PRe-medical Cancer Immunotherapy Network for Canine Trials (PRECINCT) and Nanostring Technologies and supported by a grant from the National Cancer Institute (U24-CA224122 SUPPLEMENT; PI: Nicola Mason). Genes known to be associated with intracranial gliomas in both pet dogs and humans were selected based on their relevance for the study of oncology as well as expression profiles from both RNA-Seq and nCounter experiments.
