# Supplementary material for: Evaluation of immunologic parameters in canine glioma patients treated with an oncolytic herpes virus
Source: J Transl Genet Genom. Author manuscript; Available in PMC 2022 Mar 25. (PMC8955901; doi:10.20517/jtgg.2021.31)
Supplement: supplementary materials [file NIHMS1778534-supplement-supplementary_materials.zip › supplementary materials/jtgg-2021-31-SupplementaryTable3.pdf]

Supplemental Table 3: Top 20 Differentially expressed mRNA genes in treatment naïve canine gliomas vs. normal tissue.

mRNA	Linear Fold Change	p-value	Function
DUSP6	6.17	3.52×10^{-4}	Cytokine signaling
CCND1	8.99	6.58×10^{-4}	Cell cycle
CDKN2C	4.77	7.02×10^{-4}	Cell cycle
CDK1	5.89	0.00123	Cell cycle
CDK4	3.4	0.0015	Cell cycle
NT5E	6.91	0.00163	Transporter
MK167	14.3	0.00237	Cell cycle
OLIG2	4.07	0.00279	Epigenetic Regulation
TP53	2.8	0.00303	Apoptosis
IL1RAP	5.05	0.00307	Cytokine signaling
RAD51	8.31	0.00314	Cell cycle
SOX10	5.64	0.00331	Epigenetic Regulation
RAF1	1.77	0.00415	MAPK, Senesence
NRAS	1.92	0.00537	MAPK, Senesence
MAP3K1	3.66	0.00552	MAPK, Senesence
CD276	1.93	0.00594	Immune Regulation

PDGFRA	7.83	0.00632	Angiogenesis
SMARC4	1.51	0.00654	Epigenetic Regulation
ESR1	7.52	0.00661	Epigenetic Regulation
ERBB3	5.47	0.00706	MAPK, Regulation

Differential gene expression in canine glioma tumor tissue compared to adjacent normal brain tissue collected from non-tumor bearing animals or from regions adjacent to tumor.
